# Educational interventions using digital technologies to promote parental empowerment in situations of vulnerability: a scoping review

**DOI:** 10.1590/1980-220X-REEUSP-2025-0464en

**Published:** 2026-08-14

**Authors:** Joana Rita Guarda da Venda Rodrigues, Inês Miranda Teixeira, Joana Margarida Marques de Sousa da Piedade, Joana Santos Silva, Mariana Lopes Gonçalves, Bruno Ricardo Ferreira dos Santos Gomes, Olga Maria Martins de Sousa Valentim

**Affiliations:** 1 Universidade de Lisboa, Escola Superior de Enfermagem, Centro de Investigação Inovação e Desenvolvimento em Enfermagem de Lisboa, Lisboa, Portugal.; 2 Unidade Local de Saúde de Lisboa Ocidental, Hospital de São Francisco Xavier, Unidade de Neonatologia, Lisboa, Portugal.; 3 Unidade Local de Saúde do Algarve, Hospital de Faro, Serviço de Medicina Intensiva Neonatal e Pediátrica, Faro, Portugal.; 4 Unidade Local de Saúde de São José, Hospital Dona Estefânia, Unidade de Cuidados Intensivos, Lisboa, Portugal.; 5 Unidade Local de Saúde de São José, Maternidade Dr. Alfredo da Costa, Serviço de Neonatologia, Lisboa, Portugal.; 6 Unidade Local de Saúde de São José, Hospital Dona Estefânia, Serviço de Infecciologia, Lisboa, Portugal.

**Keywords:** Parenting, Disaster Vulnerability, Digital Technology, Empowerment, Health Education

## Abstract

**Objective:**

To map educational interventions using digital technologies that promote parental empowerment in situations of vulnerability.

**Method:**

A scoping review according to the JBI methodology was conducted. Searches were carried out in the CINAHL, MEDLINE, Psychology and Behavioral Science Collection, MedicLatina, Scopus, Cochrane Central Register of Controlled Trials, Cochrane Database of Systematic Reviews, Cochrane Clinical Answers, and Web of Science databases, as well as in the Repositório Científico de Acesso Aberto de Portugal and the Google Scholar search engine. Eligibility criteria included parents and families in situations of vulnerability, digital educational interventions promoting parental empowerment, and all intervention settings.

**Results:**

984 records were identified, and ten studies were included. The evidence was distributed across four axes of vulnerability, involving participants from the gestational period to early childhood. Interventions were conducted in hospital and/or home settings, with mobile applications predominating, followed by platforms/websites, with occasional use of social media, messaging, and video resources. Information provision predominated, frequently combined with emotional regulation. Minority interventions involving behavior demonstration and environmental modification were also identified.

**Conclusion:**

Digital educational interventions appear promising and complementary to in-person care, strengthening the relationship and communication between parents and professionals and enabling more accessible, customizable, and continuous care.

## INTRODUCTION

Parenting constitutes a focus of attention in nursing care practice and is formally described as the recognition and adoption of the responsibilities inherent to being a mother and/or father. This orientation is reflected in behaviors intended to facilitate the integration of newborns (NBs) into the family unit and optimize growth and development^(1)^. It may involve progressive stages of bonding, adaptation, parental identity construction, and acceptance of the new being, resulting from a process of awareness of new responsibilities and acquisition of knowledge and skills, emerging as a situation of vulnerability^(2,3,4)^.

Although vulnerability may vary according to its associated dimensions, it is understood here as susceptibility and risk of suffering harm resulting from multidimensional processes – political, economic, cultural, psychological, or biomedical^(5)^ – constituting a dynamic and procedural phenomenon^(6)^. Nevertheless, vulnerability associated with parenting may be mitigated through emotional, social, and educational support interventions carried out by healthcare professionals and centered on families, promoting their empowerment^(6)^.

In this context, parental empowerment is understood as a process through which parents gain greater control over the decisions and actions that influence their children’s lives and health, becoming more aware of their abilities, strengthening self-confidence, acquiring knowledge, and developing skills that enable them to play an active role in promoting child development and well-being^(7)^, particularly in the cognitive, affective, and behavioral dimensions^(8)^. In this regard, nurses’ intervention is particularly relevant in the parental empowerment process, which aims to promote a healthy family environment favorable to child development. This process is facilitated by educational interventions that value parenting, promote collaborative relationships, and mobilize community resources^(8)^, and may also be consolidated through behavior change techniques, namely: (i) information provision, supplying knowledge about the health consequences of behaviors to be modified; (ii) behavior demonstration, using role-playing or visual resources; (iii) modification of the physical and social environment, favoring the adoption of the desired behavior; and (iv) emotional regulation, reducing negative emotions to facilitate performance of the desired behavior^(9)^.

In a period marked by rapid evolution, digital technologies – understood as a set of resources based on information and communication technologies, including online platforms, mobile applications, social media, and smart devices – have profoundly transformed the ways health information is accessed, produced, and shared^(10,11)^. The use of these interactive digital technologies has enabled closer collaboration between healthcare professionals and consumers, favoring the adaptation of interventions to the specific needs of individuals and families in situations of vulnerability^(10)^. Thus, healthcare environments have been reconfigured through educational interventions supported by digital technology, redefining how health education is conceived and implemented, enabling greater reach, personalization, and interactivity^(11)^. Although digital technologies do not replace traditional practices, they function as complementary resources promoting relationships and communication between parents and professionals^(12)^, contributing to strengthening parental knowledge and care^(13)^. However, despite their recognized potential for parental empowerment and self-care promotion, the systematic integration of these digital health technologies into services aimed at families in situations of vulnerability, such as during the transition to parenting, remains limited^(14)^.

A preliminary search identified ten reviews. Among these, four systematic reviews^(15,16,17,18)^, four scoping reviews^(14,19,20,21)^, one integrative review^(13)^, and one narrative review^(22)^ were found. In general, the identified reviews do not explicitly address the specific contribution of digital interventions to parental empowerment, prioritizing the assessment of the overall effectiveness of interventions in highly specific contexts, age groups, and thematic areas (namely adolescent pregnancy^(15)^, pregnancy and the postpartum period^(14)^, prenatal^(18)^, perinatal^(21)^, or neonatal periods^(16,17,18,19,20,21,22)^, breastfeeding^(17)^, preterm NBs^(13)^, and families of children with special healthcare needs^(19)^). Therefore, considering the diversity of educational interventions using digital technologies aimed at parenting, as well as the heterogeneity of populations, contexts, and technological resources identified in the literature, conducting a scoping review was considered the most appropriate methodological design, as it allows mapping the extent, variety, and nature of existing evidence, as well as comprehensively exploring digital educational interventions that promote parental empowerment in situations of vulnerability, contributing to the identification of knowledge gaps and guiding future research.

The present review therefore differs from existing reviews by focusing on mapping educational interventions using digital technologies that promote parental empowerment in situations of vulnerability. It presents an innovative character by organizing the identified interventions according to behavior change techniques, namely information provision, behavior demonstration, environmental modification, and emotional regulation. It is also particularly relevant because it broadly addresses parenting from pregnancy to early childhood (≤36 months), a critical period for child development and, therefore, a period of particular vulnerability for neurodevelopment^(23)^.

By integrating the dimension of vulnerability inherent to parenting and describing the behavior change techniques mobilized in digital educational interventions, this review fills a gap in the literature by offering a more explanatory understanding of how such interventions may promote parental empowerment, contributing to guiding digital educational practices tailored to the needs of families transitioning to and experiencing parenting.

Accordingly, this scoping review was conducted with the general objective of mapping educational interventions using digital technologies that promote parental empowerment in situations of vulnerability. The specific objectives were: (a) to identify situations of vulnerability associated with parenting addressed in the included studies; (b) to identify the contexts in which digital educational interventions and technological resources were applied; and (c) to identify the behavior change techniques used in digital educational interventions promoting parental empowerment. Considering these objectives, this review sought to answer the review question: “What interventions using digital technologies promote parental empowerment in situations of vulnerability?” and the following subquestions: “What situations of vulnerability associated with parenting are addressed in the included studies?”, “In which contexts are educational interventions using digital technologies applied?”, “What types of technological resources are used in the identified educational interventions?”, and “What behavior change techniques are used in educational interventions using digital technologies that promote parental empowerment?”.

## METHOD

This scoping review was conducted according to the JBI methodology^(24)^. The reporting of its implementation followed the consensus guidelines of the Preferred Reporting Items for Systematic Reviews and Meta-Analyses extension for Scoping Reviews (PRISMA-ScR)^(25,26)^. The study selection process was presented according to the PRISMA-ScR 2020 flowchart^(27)^. The protocol for this scoping review was developed by the authors and registered in the Open Science Framework (https://osf.io/sr6tw/overview).

### Eligibility Criteria

After defining the research problem and for the purpose of conducting the search, eligibility criteria were established based on Population, Concept, and Context (PCC). The population of this study comprised scientific publications whose population (P) included parents and families in situations of vulnerability, from the perinatal period to early childhood (≤36 months). Regarding the concept, scientific publications addressing educational interventions using digital technologies that promote parental empowerment were included. Concerning the context (C), intervention settings were considered without restriction, including healthcare institutions, foundations, or home settings.

This review included qualitative, quantitative, or mixed-methods studies (descriptive-exploratory studies, randomized and non-randomized clinical trials, and observational intervention studies) of any level of evidence. Studies were considered regardless of publication year or language.

Exclusion criteria included failure to meet the PCC criteria described above, as well as the following factors: unclear digital intervention and vulnerability framework in parenting; parents of children older than 36 months; systematic and integrative reviews; ongoing projects and other documents without published empirical results; article inaccessibility after contact with the author; and requests for support from the Documentation and Library Center.

### Search Strategy and Study Identification

To identify the studies, international electronic databases recognized for their broad coverage in the fields of health, nursing, psychology, and clinical evidence were used, aiming to maximize search sensitivity and identify relevant published literature on the phenomenon under study. Searches were conducted in the databases available through the EBSCO host platform: CINAHL Ultimate, MEDLINE Ultimate, Psychology and Behavioral Science Collection, MedicLatina, Scopus, Cochrane Central Register of Controlled Trials, Cochrane Database of Systematic Reviews, Cochrane Clinical Answers, and Web of Science. Several tests were conducted to refine the search strategy. Searches were also performed in the *Repositório Científico de Acesso Aberto de Portugal* and in the Google Scholar search engine. The first ten pages were considered because they presented the most relevant results for the search conducted. The search took place in March 2025 and was carried out in three stages.

As a starting point, a bibliographic search was conducted on the EBSCO platform using healthcare databases such as CINAHL Ultimate and Medical MEDLINE Ultimate to identify natural language terms, keywords most frequently used in titles and abstracts, and indexing terms. In the second stage, the identified natural terms, keywords, and indexing terms were combined using Boolean operators and, whenever possible, the asterisk operator (*) to form the search expression, which was adapted to the specificities of each database or repository. The combined terms used included: “parent”; “family”; “digital health”; “internet-based intervention”; “digital technology”; “health education”; “parental empowerment”; “health promotion”; “parenting education”; “neonat*”; and “hospital*”.

In the final stage, the bibliographic references present in the previously identified records were analyzed. The search also included gray literature to identify potentially relevant studies; however, only documents with published results in accordance with the predefined inclusion criteria were considered eligible for inclusion.

### Data Extraction, Analysis, and Synthesis

The analysis and selection of records were carried out using the Qatar Computing Research Institute platform (Rayyan QCRI), through which duplicate records were identified and removed.

After duplicate removal, the identified records underwent a single stage of title and abstract screening according to the JBI methodology for scoping reviews. This screening was independently conducted by four reviewers based on the previously defined inclusion and exclusion criteria.

Studies considered potentially eligible were subsequently retrieved in full and assessed in full text regarding compliance with the eligibility criteria. Disagreements between reviewers were resolved by consensus, ensuring the robustness of the selection process. Whenever the full text was unavailable, the corresponding author was contacted and/or the article was requested from a university library.

Descriptive analysis and data synthesis were independently and blindly performed by two or more reviewers using data extraction tables developed by the research team. These tables included dimensions related to the characterization of publications and selected studies, as well as categorization of interventions according to parenting-related situations of vulnerability, application context, digital technologies used, and identified behavior change techniques. This categorization was aligned with the objectives and review question. After mutual review of the records, disagreements were clarified with the support of a third reviewer, culminating in the presentation of consensual results.

## RESULTS

**Figure 1:**
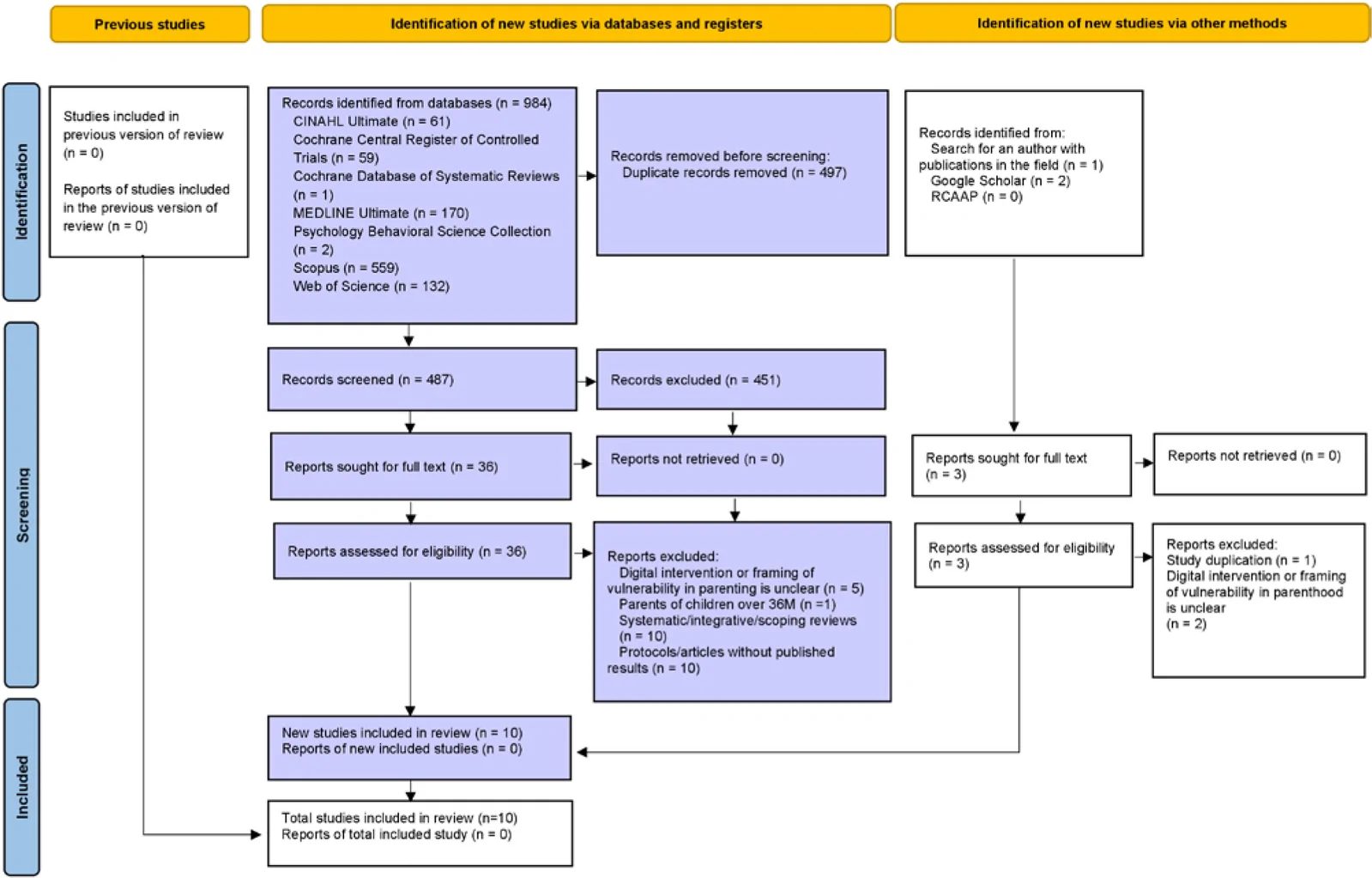
Flowchart of the process for identifying, screening, determining eligibility, and including studies – Lisbon, Portugal, 2025.

The search conducted in the databases identified a total of 984 records. Following the selection and eligibility process, ten scientific publications were included in this review. The results obtained from the screening process were presented according to the PRISMA-ScR 2020 flowchart^(27)^ (Figure 1).

The results of this scoping review were presented descriptively and organized into summary tables to provide a comprehensive overview of the identified digital educational interventions, situations of vulnerability associated with parenting, application contexts, technological resources used, and behavior change techniques mobilized. The following tables summarize the main characteristics and information of the included studies.

Chart 1 presents the general characterization of the included studies, namely publication year, title, country of origin, study type, objective, participants, and intervention moderators.

**Chart 1:** General characterization of the publications included in the review – Lisbon, Portugal, 2025.

Authors | Year | Article title	Country	Study design	Objective	Participants	Intervention moderators
Anne H. Salonen, Karen F. Pridham, Roger L. Brown, Marja Kaunonen (2014)^(28)^ Impact of an internet-based intervention on Finnish mothers’ perceptions of parenting satisfaction, infant centrality and depressive symptoms during the postpartum year	Finland	Quasi-experimental and longitudinal with a control group	Evaluates how an internet-based intervention affects mothers’ parenting satisfaction, infant centrality and depressive symptoms during the postpartum year.	760 mothers in the perinatal period	Nurses
Michael G. Sawyer, Christy E. Reece, Kerrie Bowering, Debra Jeffs, Alyssa C. P. Sawyer, Murthy Mittinty, John W. Lynch (2017)^(29)^ Nurse-Moderated Internet-Based Support for New Mothers: Non-Inferiority, Randomized Controlled Trial	Australia	Randomized controlled non-inferiority clinical trial	Test the non-inferiority of outcomes for mothers and infants who received a clinic-based postnatal health check plus nurse-moderated, Internetbased group support, compared with outcomes for those who received standard care consisting of postnatal home-based support provided by a community nurse	819 women during the postpartum period who were discharged from the hospital, with children between 1 and 7 months of age	Nurses
Deborah E. McCarter, Eugene Demidenko &. Mark T. Hegel (2018)^(30)^ Measuring outcomes of digital technology-assisted nursing postpartum: A randomized controlled trial	United States of America	Longitudinal cohort in three phases: phases 1 and 2 were feasibility and acceptability studies; phase 3 was a randomized controlled clinical trial	Determine if delivering electronic messages from nurses during the first six months postpartum is feasible, acceptable and effective in improving mood and decreasing parenting stress.	Phase 1 – 75 women during the postpartum period Phase 2 – 55 women during the postpartum period Phase 3 – 547 women during the postpartum period	Nurses
J. Banerjee, A. Aloysius, K. Platonos, & A. Deierl (2018)^(31)^ Innovations: Supporting family integrated care	United Kingdom		Analyze innovative ways to break down the barriers of education and communication, as well as parental access to the crib, through innovative ideas and digital technologies.		Nurses
Cigdem Sari & Naime Altay (2020)^(32)^ Effects of providing nursing care with web-based program on maternal self-efficacy and infant health	Turkey	Randomized clinical trial with post-test design	Examine the efficacy of a web-based program (informed by Pender’s Health Promotion Model), given to primiparous women on the growth and development of infant, infants’ health, and women’s self-efficacy level.	71 primiparous women and their babies recruited from three primary healthcare clinics	Nurses
Annica Sjöström Strand, Björn Johnsson, Momota Hena, Boris Magnusson, Inger Kristensson Hallström (2022)^(33)^ Developing eHealth in neonatal care to enhance parents’ self-management	Sweden	Qualitative	Develop an eHealth device supporting the transition from hospital to home for parents with premature infants, replacing or complementing in-person visits to healthcare services and offering a direct, secure, and effective communication channel with healthcare professionals, promoting self-care and family safety.	Three families tested the prototype while still in the hospital; in the next phase, three families tested the prototype in a hospital setting; and five families with infants born between 24–34 weeks of gestation tested the same prototype in their home environment.	Nurses
Antonio Di Mauro, Federica Di Mauro, Chiara Greco, Orazio Valerio Giannico, Francesca Maria Grosso, Maria Elisabetta Baldassarre, Manuela Capozza, Federico Schettini, Pasquale Stefanizzi, Nicola Laforgia (2021)^(34)^ In-hospital and web-based intervention to counteract vaccine hesitancy in very preterm infants’ families: a NICU experience	Italy	Quantitative observational	Evaluate both the coverage and the timeliness of routine immunizations in a cohort of preterm infants (< 33 weeks of gestational age) at 24 months of age whose families have been subjected to in-hospital and web-based interventions to counteract vaccine hesitancy.	Parents of 170 newborns born before 33 weeks of gestational age and who were in the Neonatal Intensive Care Unit, with internet access and a Facebook® profile.	Neonatal nurses and neonatologists
Lisa Militello, Emre Sezgin, Yungui Huang, Simon Lin (2021)^(35)^ Delivering Perinatal Health Information via a Voice Interactive App (SMILE): Mixed Methods Feasibility Study	United States of America	Feasibility of mixed-methods	Assess the feasibility of using voice technology to support perinatal health and baby care practices.	19 pregnant women (17 to 36 weeks of gestation)	
Joelle Yan Xin Chua, Mahesh Choolani, Cornelia Yin Ing Chee, Huso Yi, Yiong Huak Chan, Joan Gabrielle Lalor, Yap Seng Chong, Shefaly Shorey (2024)^(36)^ Parents’ Perceptions of Their Parenting Journeys and a Mobile App Intervention (Parentbot-A Digital Healthcare Assistant): Qualitative Process Evaluation	Singapore	Qualitative and descriptive	Explore the perinatal experiences of parents in Singapore, as well as examine the user experiences of the mobile app-based intervention with an in-built chatbot titled Parentbot-a Digital Healthcare Assistant (PDA).	118 heterosexual couples over 21 years of age recruited through purposive sampling at a tertiary hospital in Singapore	
Sahiti Myneni, Angela Ross, Alexandra Zingg, Tavleen Singh, Amy Franklin, Deevakar Rogith, Jerrie Refuerzo (2024)^(37)^ Digital health technologies for high-risk pregnancy management: three case studies using Digilego framework	United States of America	Mixed-methods	Develop and assess three digital solutions to support women with high-risk pregnancies (gestational diabetes, hypertension, and perinatal depression) through the Digilego framework.	Study 1: 9,364 women interacted with two pregnancy apps: “What to Expect” and “Baby Center” Study 2: Ten pregnant women tested “MyPregnancyChart” Study 3: Thirty pregnant women tested “MomMind”	

The ten scientific publications included in this scoping review corresponded to the studies that fully met the predefined eligibility criteria and resulted from the identification, selection, and inclusion process presented in the PRISMA-ScR flowchart. These studies were published between 2013 and 2024, corresponding to the publication period of studies conducted in eight different countries, namely the United States of America (three studies)^(30,35,37)^, Sweden (one study)^(33)^, Singapore (one study)^(36)^, Italy (one study)^(34)^, Australia (one study)^(29)^, Turkey (one study)^(32)^, Finland (one study)^(28)^, and the United Kingdom (one study)^(31)^.

The included publications were individually classified, resulting in two qualitative studies^(33,36)^, one quantitative observational study^(34)^, one mixed-methods feasibility study^(35)^, one three-phase longitudinal cohort study^(30)^ – phases 1 and 2 consisted of feasibility and acceptability studies, while phase 3 was a randomized controlled clinical trial –, one mixed-methods study^(37)^, one longitudinal quasi-experimental study^(28)^, one non-inferiority randomized controlled trial^(29)^, and one randomized clinical trial with a post-test design^(32)^.

After full analysis of the selected studies, educational interventions using digital technologies that promote parental empowerment aimed at parents in situations of vulnerability were identified. Most of the identified interventions were moderated by nurses, with one of these interventions also being moderated by neonatologists in partnership with nurses working in the Neonatal Intensive Care Unit (NICU) where the study was conducted.

Data analysis allowed the identification of five emerging categories: Situations of vulnerability in parenting; Application context of educational interventions using digital technologies; Digital technology in use; Educational interventions using digital technologies; and Behavior change techniques in interventions promoting parental empowerment. The results of this analysis are presented in Chart 2.

**Chart 2:** Summary of studies included in the literature review – Lisbon, Portugal, 2025.

Citation | Year	Situations of vulnerability in parenting	Application context of educational interventions using digital technologies	Digital technology in use	Educational interventions using digital technologies	Behavior change techniques in interventions promoting parental empowerment
Salonen *et al.*, 2014 ^(28)^	Transition to parenting (prenatal and postnatal periods) – primiparous and multiparous women during pregnancy and up to one year postpartum	Home setting	Website	The website-based intervention was designed to strengthen parental satisfaction and parental self-efficacy by offering online support regarding parenting, breastfeeding, and infant care. The website consisted of: (a) an information database; (b) an online peer discussion forum; and (c) a question-and-answer service, focusing on parental satisfaction, infant centrality, and depressive symptoms.	Information provision and environmental modification [social]
Sawyer *et al.*, 2017^(29)^	Postpartum period and adaptation to parenting – postpartum mothers (up to seven months postpartum)	Home setting (from 1 to 7 months of the child’s age)	Website	Through the website, accessible via computer or mobile phone, mothers were able to communicate with one another through a forum, asking questions about their difficulties, clarifying doubts, and mutually supporting each other. Communication among mothers was moderated by a nurse, who provided clarification based on scientific evidence.	Information provision and environmental modification [social]
McCarter *et al.*, 2018^(30)^	Postpartum period and adaptation to parenting – postpartum mothers (up to six months postpartum)	Initially in the hospital setting and subsequently at home	Text messages and telephone calls	Through educational messaging, information regarding maternal self-care and childcare was provided. In addition to informational content, a nurse experienced in maternal-child health sent inspirational and supportive messages. Group I — four messages per week for six months; Group II — four messages per week, two of which offered the possibility of replying and requesting a telephone call with a nurse during the sixmonth period.	Information provision and emotional regulation
Banerjee *et al.*, 2018^(31)^	Parenting in the context of neonatal hospitalization – parents of newborns hospitalized in the Neonatal Intensive Care Unit	Hospital setting Digital mobile application suitable for use in a home setting.	*Integrated Family Delivered Care (IFDC) digital mobile application ^**^Noise-canceling headphones ^**^Fingerprint access to the Neonatal Intensive Care Unit ^***^Video messaging system	Through the mobile application, parents were able to access extensive educational content and share their parenting journey with preterm newborns. The use of noise-canceling headphones ensured privacy for newborns and families in the Neonatal Intensive Care Unit during shift handovers, reducing interruptions in bonding with their newborns during these periods. Through biometric fingerprint access, parents were able to have continuous access to the Neonatal Intensive Care Unit. By sharing video messages between healthcare professionals and parents, frequent updates regarding the condition of newborns admitted to the Neonatal Intensive Care Unit were provided, facilitating bonding when parental presence was not possible	*Information provision, *behavior demonstration, ^**^environmental modification [physical], emotional regulation, and ^***^emotional regulation
Sari & Altay, 2020^(32)^	Transition to parenting (prenatal and postnatal periods) – prenatal phase: pregnant women between 32 and 37 weeks of gestation; postnatal phase: up to three months postpartum	The website was introduced during the prenatal phase in the PHC setting and used throughout pregnancy and the postpartum period at home	Website	Through the website, which was divided into four topics according to Pender’s Health Promotion Model – “My Baby’s Nutrition,” “My Baby’s Bath and Care,” “My Baby’s Sleep and Safety,” and “Communication with My Baby” – informational content regarding these topics was provided. In addition, through this website, parents had access to shared videos containing information and demonstrations related to the different topics, motivational messages, and contact with healthcare professionals.	Information provision, emotional regulation and behavior demonstration
Strand *et al.,* 2022^(33)^	Parenting in the context of neonatal hospitalization – parents of preterm infants (24 to 34 weeks of gestational age) transitioning to hospital discharge	The digital intervention under development was applied both in the hospital setting and in the home setting	eHealth mobile application adapted for Android tablets	Through the mobile application, nurses from the Neonatal Intensive Care Unit were able to communicate with parents through text messages, photographs, and video calls. Additionally, through the mobile application, it was possible to record and monitor the infant’s clinical data (weight, oxygen saturation levels, food intake), allowing nurses to provide remote follow-up. By using this application, healthcare professionals were also able to observe and assess the infant through video calls.	Information provision and emotional regulation
Di Mauro *et al*., 2021^(34)^	Parenting in the context of neonatal hospitalization – parents of preterm newborns up to 2 years of age	Dissemination during PHC consultations at 2, 4, 6, and 12 months of life and use in the home setting	Facebook® page	Through the Facebook® page, parents were able to access clear and simple content regarding the risks and benefits of vaccines, vaccine-preventable diseases, and the ages recommended for vaccination according to the Italian vaccination schedule.	Information provision
Militello *et al.,* 2021^(35)^	Transition to parenting (prenatal and postnatal periods) – women and families in the perinatal phase between 17 and 36 weeks of gestation	Home setting	Interactive voice mobile application called SelfManagement Intervention–Life Essentials (SMILE)	Through the mobile application, information was provided through sequential podcasts lasting three to eight minutes about infant needs and care, postpartum care and postpartum depression, infant feeding/breastfeeding, and strategies for managing marital relationships, focusing on perinatal education. At the end of each mini-podcast, parents provided feedback through audio recordings. Additionally, links to reliable documents and a list of useful contacts for accessing professional resources were provided.	Information provision
Chua *et al.*, 2024^(36)^	Transition to parenting (prenatal and postnatal periods) – parents during the perinatal and postnatal periods (from 24 weeks of gestation to one month postpartum)	Intervention introduced in the hospital setting and applied in the home setting	A mobile digital application with a chatbot called “Parentbot” – a digital health assistant (PDA)	Through the digital mobile application, parents were provided with informational, psychological, and social support during pregnancy (> 24 weeks of gestation) until the first month after delivery. The aforementioned support is provided through: – Educational materials – Discussion/support forum; – Activities for emotional regulation; – Integrated chatbot.	Information provision, emotional regulation and environmental modification [social]
Myneni *et al.*, 2024^(37)^	Perinatal phase in women with high-risk pregnancies (gestational diabetes, hypertension, and peripartum depression)	Used during pregnancy in an outpatient setting	Two digital applications: *”MyPregnancyChart” and ^**^”MomMind”	Through the MyPregnancyChart app, pregnant women can address various topics in an online discussion forum and record clinical data, allowing the system to signal when medical assessment is advisable, thus addressing monitoring, maintenance, and/or behavioral change. Through the MomMind app, mothers have access to a repository categorized by topic that helps identify symptoms of depression, teaches strategies for coping with stress, sadness, or anxiety, and recognizes and normalizes emotional difficulties felt in the peripartum period.	*Environmental modification [social], ^**^information provision, and ^**^emotional regulation

**Figure 2:**
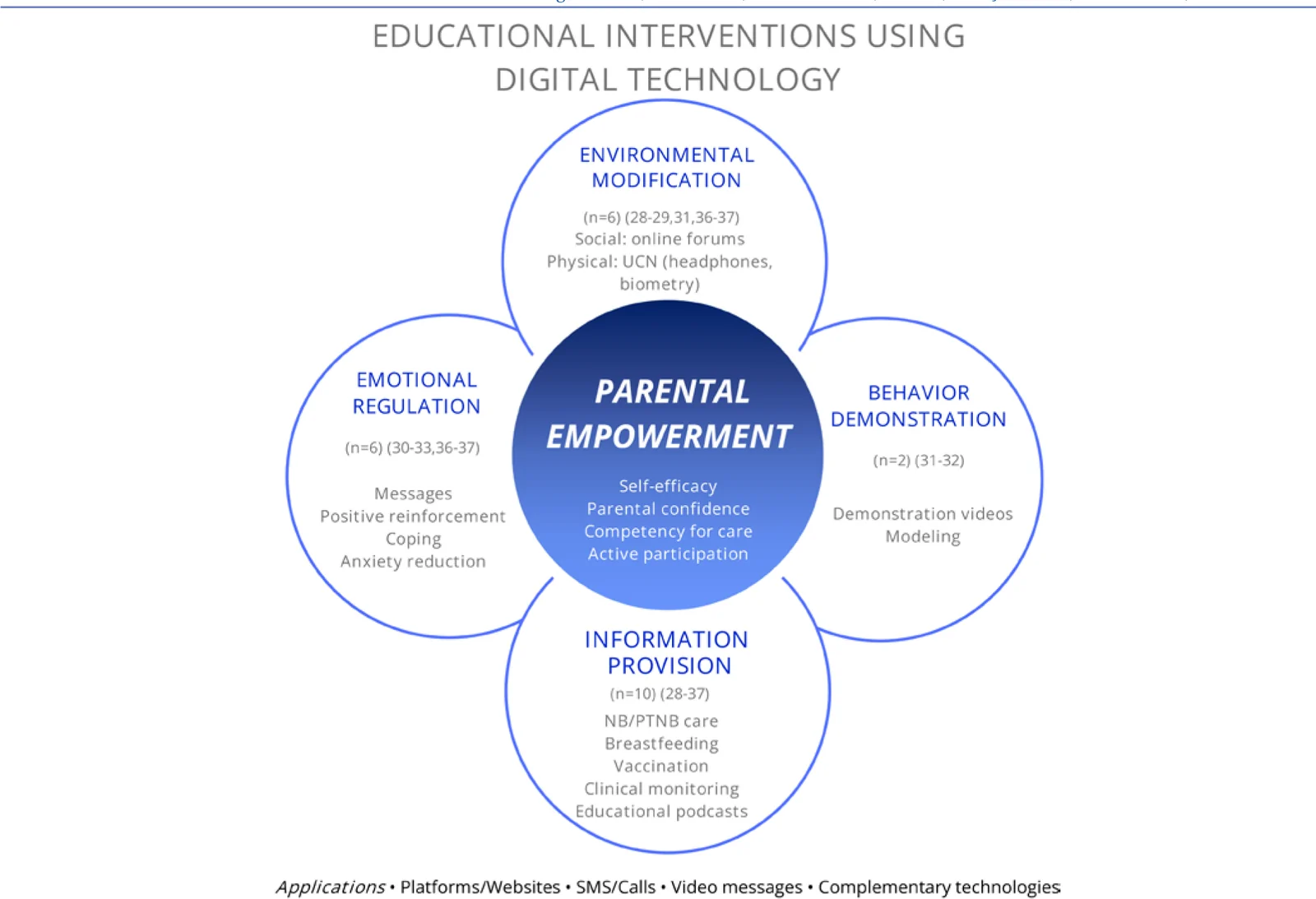
Behavioral change techniques used in digital educational interventions and their contribution to parental empowerment in vulnerable situations – Lisbon, Portugal, 2025.

To further enhance the analytical clarity of this scoping review, educational interventions using digital technologies were systematized through four behavioral change techniques – information provision, behavior demonstration, environmental modification, and emotional regulation – highlighting how these elements contribute to parental empowerment in vulnerable situations, as shown in Figure 2.

## DISCUSSION

### Situations of Vulnerability in Parenting

Within the scope of this scoping review, vulnerability in parenting is understood as a dynamic and multidimensional condition resulting from the interaction among individual, clinical, psychosocial, and contextual factors that may compromise parents’ ability to exercise parenting in an informed, safe, and confident manner. These situations include parents and families who, at certain moments of the life cycle or in specific healthcare contexts, face increased demands, uncertainty, emotional stress, or limitations in access to resources and professional support.

The included studies demonstrate that vulnerability in parenting emerges mainly during periods of transition and adaptation, as well as in complex clinical contexts in which parents are confronted with new responsibilities, critical decisions, and intensive learning needs.

The situations of vulnerability described in the included studies focus on four axes: (1) transition to parenting (prenatal and postnatal periods); (2) postpartum and adaptation to parenting; (3) high-risk pregnancy and maternal clinical vulnerability; and (4) parenting in the context of neonatal hospitalization. Overall, the populations encompassed the gestational, postpartum, and neonatal periods, extending to early childhood up to 36 months of age, a phase recognized as critical for child development and for the consolidation of parental competencies.

Regarding the transition to parenting, with emphasis on the prenatal and postnatal periods, four studies focused on the gestational period: women between 17 and 36 weeks of gestation^(35)^; parents from 24 weeks onward^(36)^; and pregnant women between 32 and 37 weeks of gestation^(32,37)^. In these contexts, vulnerability may be associated with anticipation of the parenting role, the need to acquire knowledge and competencies, and the management of expectations and emotions related to impending parenthood.

Concerning the postpartum period and adaptation to parenting, five studies included the postnatal period^(28,29,30,31,32,36)^, with follow-up extending up to one month^(36)^, six months^(30)^, and one year^(28)^. Two studies focused specifically on mothers up to three months^(29)^ and seven months postpartum^(32)^. In these cases, vulnerability may emerge from physical and emotional overload, difficulties adapting to the new role, and the need for continued support in managing NB care.

Only one study focused on high-risk pregnancy, specifically targeting women with hypertension/gestational diabetes and peripartum depression^(37)^.

Regarding parenting in the context of neonatal hospitalization, three studies were identified involving: parents of NBs admitted to the NICU^(31)^; parents of preterm infants (24–34 weeks) transitioning to discharge^(33)^; and parents of preterm NBs up to 2 years of age^(34)^. Together, these studies seem to demonstrate prolonged vulnerability associated with prematurity, hospitalization, and continuity of care after discharge, characterized by anxiety, the need for specialized information, and support in acquiring parental competencies within highly technological settings.

### Context of Application of Educational Interventions Using Digital Technologies

In line with the objective of this scoping review to map the contexts in which digital educational interventions promoting parental empowerment are applied, the results reveal that these interventions are developed across different settings, reflecting the diversity of family needs and the flexibility of these tools in adapting to different environments.

The included studies describe interventions using digital technologies presented within healthcare settings and used by the target population in hospital and/or home settings. For instance, three studies describe interventions using digital technologies that, although initiated in hospital settings, were also designed for home use, promoting continuity of care beyond discharge^(30,31,33,36)^. One of these studies describes not only the possibility of home use of the IFDC application, but also exclusively hospital-based solutions, such as noise-canceling headphones and biometric access to the NICU^(31)^.

Other studies describe educational interventions designed for home use, although introduced in clinical settings. Examples include interventions introduced by hospital teams^(30,36)^, in PHC settings^(32,34)^, or in outpatient consultations^(37)^ for subsequent use by parents at home. These findings suggest the potential of digital technologies as mediators of transition between care settings. In particular, one study reported dissemination of the technology during consultations conducted at two, four, six, and 12 months of a child’s life^(34)^.

### Educational Interventions Using Digital Technologies

The included studies addressed several digital technologies as resources to promote parental empowerment in contexts of vulnerability. Applications emerged as the most frequent resource, being present in five studies^(29,30,31,33,35,36,37)^, with functionalities ranging from eHealth^(33)^, voice assistant technology^(35)^, parenting support chatbots^(36)^, support for families in the NICU^(31)^, and applications providing informational and emotional support for mothers^(37)^.

The second most frequently mobilized digital technologies in the included studies were digital platforms and websites^(28,29,32,34)^. One Facebook^®^ page^(34)^ and educational websites with intuitive navigation^(28,29,32)^ were highlighted. This diversity of technological resources demonstrates different strategies for access to information and parental support depending on the context and characteristics of the target population.

Some studies also used other digital technologies, such as text messaging, telephone calls^(30)^, or video messaging systems to facilitate communication between parents and the healthcare team^(31)^. Two complementary digital technologies identified during this process were unrestricted parental access to the NICU through fingerprint recognition and the use of noisecanceling headphones^(31)^. Despite the diversity of technological resources, the studies converge in emphasizing accessibility, interactivity, and personalization as key elements for the success of digital interventions supporting parenting in contexts of vulnerability.

### Educational Interventions Using Digital Technologies to Promote Parental Empowerment in Situations of Vulnerability

The educational interventions using digital technologies aimed at promoting parental empowerment in situations of vulnerability identified in this review were classified into four categories corresponding to the applied behavior change techniques: (i) information provision; (ii) behavior demonstration; (iii) environmental modification; and (iv) emotional regulation^(9)^.

Most interventions were grouped within information provision, i.e., interventions centered on promoting parental knowledge regarding essential concepts and themes to be developed and acquired in parenting^(28,29,30,31,32,33,34,35,36,37)^. Among these interventions, only two presented information provision as the sole and exclusive strategy for promoting parental empowerment^(34,35)^.

Four of the identified studies combined information provision with emotional regulation, using strategies designed to reduce anxiety/fear and strengthen self-efficacy^(30,32,36,37)^. In another study, a predominantly informational intervention appeared to contribute indirectly and consequentially to parents’ emotional regulation, with parents reporting greater self-efficacy, security, and confidence in parental performance^(33)^. These findings highlight the interrelationship between informational and emotional components in the parental empowerment process.

A study describes a digital intervention that promotes parental empowerment exclusively through emotional regulation, using an online platform to send informational and positive reinforcement messages, in addition to multimedia content regarding the NB’s condition and development, enabling remote monitoring of significant milestones^(31)^.

Behavior demonstration emerged in two interventions – a website^(32)^ and the “Integrated Family Delivered Care” application^(31)^ – both of which provided demonstrative care videos in addition to informational content and emotional regulation components.

It is noteworthy that six of the 14 interventions promoted parental empowerment through environmental modification: four at the level of the social environment^(28,29,36,37)^ – through the creation of online discussion forums among parents – and two within the physical environment – through the use of noisecanceling headphones^(31)^ to remain in the NICU and fingerprint access^(31)^ to the unit. These strategies also contributed to emotional regulation (reduction of separation anxiety and distress associated with access difficulties).

In summary, the results seem to demonstrate that while some interventions operate through a single behavioral component, others combine multiple categories, frequently interrelated and with consequential effects on parental empowerment, responding differently to the identified situations of vulnerability.

## CONCLUSION

This scoping review made it possible to map educational interventions using digital technologies aimed at promoting parental empowerment in situations of vulnerability, highlighting the diversity of contexts, technological resources, and behavior change techniques mobilized throughout the transition to and experience of parenting.

The results suggest that digital technologies constitute particularly relevant resources in contexts of vulnerability because they allow flexible, continuous, and personalized responses to parents’ informational and emotional needs, especially during the prenatal and postnatal periods and in neonatal hospitalization settings. In this regard, parental empowerment emerges not only as an expected outcome, but also as a dynamic process supported by the combination of evidence-based information, emotional support, and modification of the care environment.

The identified digital educational interventions were mostly mediated by nurses, reinforcing the role of these professionals as central agents in promoting parenting, health literacy, and parental autonomy, in line with a family-centered approach. The integration of these interventions seems to be able to broaden the scope of educational intervention beyond in-person contact, favoring continuity of care and strengthening the relationship and communication between parents and healthcare professionals.

The main contribution of this review lies in the organization of digital educational interventions according to behavioral change techniques, allowing for a more explanatory reading of the mechanisms through which these interventions can promote parental empowerment in vulnerable situations.

Despite the identified potential, relevant gaps persist in the literature, namely the scarcity of studies with experimental or quasi-experimental design, the limited assessment of the medium- and long-term impact of the interventions, and the reduced exploration of the perspective of healthcare professionals involved in their implementation. It is suggested that future research should prioritize the development and assessment of digital interventions co-designed with parents and professionals, integrating multiple behavioral change techniques and assessing outcomes focused on parental empowerment and transition processes in and to parenthood.

This review did not include an assessment of the methodological quality of the studies, in accordance with the JBI methodology for scoping reviews, which limits inferences about the robustness of the effects. Although a comprehensive search was conducted in multiple databases, the absence of a systematic search in international databases of theses and dissertations and the limitation of the search in Google Scholar to the first ten pages of results may have conditioned the identification of all potentially relevant evidence. These aspects reinforce the need for methodologically rigorous primary studies and future systematic reviews that further assess the effectiveness of digital educational interventions that promote parental empowerment.

## Data Availability

The entire dataset supporting the results of this study was published in the article itself.
