# Supplementary material for: Educational interventions using digital technologies to promote parental empowerment in situations of vulnerability: a scoping review
Source: Rev Esc Enferm USP. 2026 Aug 14;60:e20250464. doi: 10.1590/1980-220X-REEUSP-2025-0464en (PMC13544914; doi:10.1590/1980-220X-REEUSP-2025-0464en)
Supplement: Translation

# Translation: Intervenções educativas com recurso a tecnologias digitais promotoras do empowerment parental em situações de vulnerabilidade: scoping review

**DOI:** 10.1590/1980-220X-REEUSP-2025-0464pt

**Authors:** Joana Rita Guarda da Venda Rodrigues, Inês Miranda Teixeira, Joana Margarida Marques de Sousa da Piedade, Joana Santos Silva, Mariana Lopes Gonçalves, Bruno Ricardo Ferreira dos Santos Gomes, Olga Maria Martins de Sousa Valentim

**Affiliations:** 1 Universidade de Lisboa, Escola Superior de Enfermagem, Centro de Investigação Inovação e Desenvolvimento em Enfermagem de Lisboa, Lisboa, Portugal.; 2 Unidade Local de Saúde de Lisboa Ocidental, Hospital de São Francisco Xavier, Unidade de Neonatologia, Lisboa, Portugal.; 3 Unidade Local de Saúde do Algarve, Hospital de Faro, Serviço de Medicina Intensiva Neonatal e Pediátrica, Faro, Portugal.; 4 Unidade Local de Saúde de São José, Hospital Dona Estefânia, Unidade de Cuidados Intensivos Neonatais, Lisboa, Portugal.; 5 Unidade Local de Saúde de São José, Maternidade Dr. Alfredo da Costa, Serviço de Neonatologia, Lisboa, Portugal.; 6 Unidade Local de Saúde de São José, Hospital Dona Estefânia, Serviço de Infecciologia, Lisboa, Portugal.

**Keywords:** Poder Familiar, Vulnerabilidade a Desastres, Tecnologia Digital, Empoderamento, Educação em Saúde

## Abstract

**Objetivo:**

Mapear as intervenções educativas com recurso a tecnologia digital promotoras do empowerment parental em situações de vulnerabilidade.

**Método:**

Scoping review segundo a metodologia JBI. Realizou-se pesquisa nas bases CINAHL, MEDLINE, Psychology and Behavioral Science Collection, MedicLatina, Scopus, Cochrane Central Register of Controlled Trials, Cochrane Database of Systematic Reviews, Cochrane Clinical Answers e Web of Science, bem como no portal Repositório Científico de Acesso Aberto de Portugal e no motor de busca Google Scholar. Os critérios de elegibilidade contemplaram pais e famílias em situação de vulnerabilidade, intervenções educativas digitais promotoras do empowerment parental e todos os contextos de intervenção.

**Resultados:**

Identificaram-se 984 registos, e incluíram-se dez estudos. A evidência foi distribuída em quatro eixos de vulnerabilidade, envolvendo participantes desde o período gestacional até a primeira infância. As intervenções ocorreram em contextos hospitalares e/ou domiciliários, predominando aplicações móveis, seguidas de plataformas/websites, com recurso pontual de redes sociais, mensagens e vídeo. Predominou a transmissão de informação, frequentemente combinada com regulação emocional. Identificaram-se intervenções minoritárias de demonstração do comportamento e modificação do ambiente.

**Conclusão:**

Intervenções educativas digitais mostram-se promissoras e complementares ao presencial, reforçando a relação e a comunicação entre pais e profissionais e possibilitando cuidados mais acessíveis, personalizáveis e continuados.

## INTRODUÇÃO

A parentalidade constitui um foco de atenção na prática de cuidados de enfermagem, encontrando-se formalmente descrita como o reconhecimento e a adoção das responsabilidades inerentes a ser mãe e/ou pai. Esta orientação traduz-se em comportamentos destinados a facilitar a integração do recém-nascido (RN) na unidade familiar e otimizar o seu crescimento e desenvolvimento^(1)^. Esta pode envolver etapas progressivas de vinculação, adaptação, construção da identidade parental e aceitação do novo ser, resultante de um processo de consciencialização de novas responsabilidades, aquisição de conhecimentos e competências, emergindo como uma situação de vulnerabilidade^(2,3,4)^.

A vulnerabilidade, apesar de poder assumir variações consoante as diferentes dimensões que lhe estão associadas, é aqui entendida como a suscetibilidade e o risco de se sofrer danos a partir de processos multidimensionais – políticos, económicos, culturais, psicológicos ou biomédicos^(5)^ -, constituindo um fenómeno dinâmico e processual^(6)^. Não obstante, a vulnerabilidade associada ao exercício da parentalidade pode ser atenuada através de intervenções de apoio emocional, social e educacional encetadas por profissionais de saúde e centradas nas famílias promotoras do seu *empowerment* ^(6)^.

Nesse contexto, o *empowerment* parental é compreendido como um processo através do qual os pais ganham maior controlo sobre as decisões e ações que influenciam a vida e a saúde dos filhos, tornando-se mais conscientes das suas capacidades, fortalecendo a autoconfiança, adquirindo conhecimentos e desenvolvendo competências que lhes permitem exercer um papel ativo na promoção do desenvolvimento infantil e do seu bem-estar^(7)^, nomeadamente nas dimensões cognitivas, afetivas e comportamentais^(8)^. Nesse âmbito, a intervenção do enfermeiro é particularmente relevante no processo de empoderamento parental, que visa promover um ambiente familiar saudável e favorável ao desenvolvimento infantil. Esse processo é facilitado por intervenções educativas que valorizam a parentalidade, promovem relações colaborativas e mobilizam recursos comunitários^(8)^, podendo ser ainda consolidado por técnicas de mudança comportamental, entre as quais se destacam: (i) transmissão de informação, fornecendo conhecimento sobre as consequências para a saúde de comportamentos a modificar; (ii) demonstração do comportamento, recorrendo à encenação ou recursos visuais; (iii) modificação do ambiente físico e social, favorecendo a adoção do comportamento desejado; (iv) regulação emocional, reduzindo emoções negativas para facilitar o desempenho do comportamento desejado^(9)^.

Em um período marcado pela rápida evolução, as tecnologias digitais – entendidas como um conjunto de recursos baseados em tecnologias de informação e comunicação, incluindo plataformas *online*, aplicações móveis, redes sociais e dispositivos inteligentes – têm transformado profundamente os modos de acesso, produção e partilha de informação em saúde^(10,11)^. A utilização dessas tecnologias digitais interativas tem permitido uma colaboração mais próxima entre profissionais de saúde e consumidores, favorecendo a adaptação das intervenções às necessidades específicas de indivíduos e famílias em situação de vulnerabilidade^(10)^. Assim, os ambientes de saúde têm vindo a ser reconfigurados por meio de intervenções educativas apoiadas na tecnologia digital, que redefinem a forma como a educação em saúde é concebida e implementada, possibilitando um maior alcance, uma maior personalização e uma maior interatividade^11)^. Embora as tecnologias digitais não substituam as práticas tradicionais, funcionam como recursos complementares promotores da relação e comunicação entre pais e profissionais^(12)^, contribuindo para o reforço do conhecimento e dos cuidados parentais^(13)^. Todavia, apesar do seu reconhecido potencial para o *empowerment* parental e para a promoção do autocuidado, a integração sistemática dessas tecnologias digitais em saúde nos serviços dirigidos a famílias em situação de vulnerabilidade, como na transição para a parentalidade, ainda é limitada^(14)^.

Foi realizada uma pesquisa preliminar, tendo sido identificadas dez revisões. Entre essas, encontram-se quatro revisões sistemáticas^(15,16,17,18)^, quatro *scoping reviews* ^(14,19,20,21)^, uma revisão integrativa^(13)^ e uma revisão narrativa^(22)^. Na generalidade, as revisões identificadas não explicitam o contributo específico das intervenções digitais para o *empowerment* parental, privilegiando a avaliação de eficácia global das intervenções em contextos, faixas etárias e áreas temáticas muito específicos (designadamente, gravidez em adolescentes^(15)^, gravidez e período pós-natal^(14)^, períodos pré-natal^(18)^, perinatal^(21)^ou neonatal^(16,17,18,19,20,21,22)^, amamentação^(17)^, RNs prematuros^(13)^ e famílias com crianças com necessidades especiais de cuidados de saúde^(19)^). Assim, atendendo à diversidade das intervenções educativas com recurso a tecnologias digitais dirigidas à parentalidade, bem como à heterogeneidade das populações, contextos e recursos tecnológicos identificados na literatura, a realização de uma *scoping review* constituiu o desenho metodológico mais apropriado, uma vez que permite mapear a extensão, a variedade e a natureza da evidência existente, bem como explorar, de forma abrangente, as intervenções educativas digitais promotoras do *empowerment* parental em situações de vulnerabilidade, contribuindo para a identificação de lacunas de conhecimento e para a orientação de futuras investigações.

A presente revisão distingue-se, assim, das revisões existentes por se centrar no mapeamento de intervenções educativas com recurso a tecnologias digitais promotoras do *empowerment* parental em situações de vulnerabilidade. Apresenta um carácter inovador pela organização das intervenções identificadas segundo técnicas de mudança comportamental, nomeadamente transmissão de informação, demonstração do comportamento, modificação do ambiente e regulação de emoções. Assume ainda particular relevância por abranger transversalmente a parentalidade desde a gestação até a primeira infância (≤36 meses), período crítico para o desenvolvimento infantil e, portanto, de particular vulnerabilidade para o neurodesenvolvimento^(23)^.

Ao integrar a dimensão da vulnerabilidade inerente à parentalidade e ao descrever as técnicas de mudança comportamental mobilizadas nas intervenções educativas digitais, esta revisão preenche uma lacuna na literatura ao oferecer uma leitura mais explicativa do como as referidas intervenções podem promover o *empowerment* parental, contribuindo para orientar práticas educativas digitais ajustadas às necessidades de famílias em transição para e na parentalidade.

Nesse sentido, realizou-se a presente *scoping review*, que tem como objetivo geral mapear as intervenções educativas com recurso a tecnologias digitais promotoras do *empowerment* parental em situações de vulnerabilidade. Os objetivos específicos foram: (a) identificar as situações de vulnerabilidade associadas à parentalidade abordadas nos estudos incluídos; (b) identificar os contextos de aplicação das intervenções educativas digitais e os recursos tecnológicos mobilizados; (c) identificar as técnicas de mudança comportamental utilizadas nas intervenções educativas digitais promotoras do *empowerment* parental.

Considerando esses objetivos, pretende-se dar resposta à questão de revisão “Quais são as intervenções com recurso a tecnologias digitais que promovem o *empowerment* parental em situações de vulnerabilidade?” e às subquestões “Quais são as situações de vulnerabilidade associadas à parentalidade abordadas nos estudos incluídos?”, “Em que contextos são aplicadas as intervenções educativas com recurso a tecnologias digitais?”, “Quais tipos de recursos tecnológicos são utilizados nas intervenções educativas identificadas?” e “Quais são as técnicas de mudança comportamental utilizadas nas intervenções educativas com recurso a tecnologias digitais promotoras do *empowerment* parental?”.

## MÉTODO

A presente *scoping review* foi realizada de acordo com a metodologia JBI^(24)^. A redação de sua implementação seguiu as diretrizes de consenso do *checklist Preferred Reporting Items for Systematic reviews and Metanalyses extension for Scoping Reviews* (PRISMA-ScR)^(25,26)^. O processo de seleção de estudos foi apresentado de acordo com o fluxograma PRISMA-ScR 2020^(27)^. O protocolo desta *scoping review* foi desenvolvido pelos autores e registado no *Open Science Framework* (https://osf.io/sr6tw/overview).

### Critérios de Elegibilidade

Após a delimitação da problemática e para a realização da pesquisa, foram definidos os critérios de elegibilidade com base na População, Conceito e Contexto (PCC). A população deste estudo integra publicações científicas cuja população (P) são os pais e famílias em situação de vulnerabilidade, desde o período perinatal até a primeira infância (≤ 36 meses). No que diz respeito ao conceito, pretendeu-se incluir publicações científicas que abordassem as intervenções educativas com recurso a tecnologias digitais promotoras do *empowerment* parental. Relativamente ao contexto (C), foram considerados contextos de intervenção sem qualquer limitação, como instituições de saúde, fundações ou domiciliar.

Nesta revisão, foram incluídos estudos qualitativos, quantitativos ou mistos (descritivo-exploratórios, ensaios clínicos randomizados e não randomizados, estudos de intervenção observacionais) de qualquer nível de evidência. Os estudos foram considerados independentemente do ano de publicação e do idioma.

Como critérios de exclusão, considerou-se o incumprimento dos critérios de PCC descritos anteriormente, bem como os seguintes fatores: a intervenção digital e o enquadramento da vulnerabilidade na parentalidade não serem claros; pais de crianças com mais de 36 meses; revisões sistemáticas e integrativas; projetos em desenvolvimento e outros documentos sem resultados empíricos publicados; inacessibilidade do artigo após solicitação ao autor; e pedido de apoio ao Centro de Documentação e Biblioteca.

### Estratégia de Pesquisa e Identificação dos Estudos

Para a identificação dos estudos, foram utilizadas as bases de dados eletrónicas internacionais reconhecidas pela sua ampla cobertura nas áreas da saúde, enfermagem, psicologia e evidência clínica, com o objetivo de maximizar a sensibilidade da pesquisa e identificar a literatura publicada relevante para o fenómeno em estudo. Foram pesquisadas as bases da plataforma *EBSCO host: CINAHL Ultimate*, *MEDLINE Ultimate*, *Psychology and Behavioral Science Collection*, *MedicLatina*, Scopus, *Cochrane Central Register of Controlled Trials, Cochrane Database of Systematic Reviews, Cochrane Clinical Answers* e *Web of Science*. Foram realizados vários testes para refinar a estratégia de pesquisa. Também foi realizada a pesquisa no portal Repositório Científico de Acesso Aberto de Portugal e no motor de busca *Google Scholar*. Foram consideradas as primeiras dez páginas por serem aquelas que apresentam os resultados mais relevantes diante da pesquisa efetuada. A pesquisa ocorreu em março de 2025 e realizou-se em três etapas.

Como ponto de partida, foi realizada uma pesquisa de bibliografia na plataforma *EBSCO*, recorrendo a bases de dados da área da saúde, como a *CINAHL Ultimate* e a *Medical MEDLINE Ultimate*, com a finalidade de identificar as palavras em linguagem natural e as palavras-chave utilizadas com maior frequência nos títulos e resumos, bem como os termos de indexação. Na segunda etapa, as palavras naturais, as palavras-chave e os termos de indexação identificados foram combinados com operadores booleanos e, sempre que possível, com o operador asterisco (*) para formar a expressão de pesquisa, que foi adaptada às especificidades de cada base de dados ou repositório. Os termos combinados utilizados incluíram: “parent*”; “*family*”; “*digital health*”; “*internet-based intervention*”; “*digital technology*”; “*health education*”; “*parental empowerment*”; “*health promotion*”; “*parenting education*”; “neonat*”; e “hospital*”.

Na última etapa, as referências bibliográficas presentes nos registos previamente identificados foram analisadas. A pesquisa incluiu igualmente literatura cinzenta, com o objetivo de identificar estudos potencialmente relevantes; contudo, apenas foram considerados elegíveis para inclusão documentos com resultados publicados em conformidade com os critérios de inclusão definidos.

### Extração, Análise e Síntese dos Dados

A análise e seleção dos registos foi realizada com recurso à plataforma *Qatar Computing Research Institute (Rayyan QCRI)*, através da qual foram identificados e removidos os registos duplicados.

Após a remoção de duplicados, os registos identificados foram submetidos a uma fase única de triagem de títulos e resumos, de acordo com a metodologia JBI para *scoping reviews*. Essa triagem foi realizada de forma independente por quatro revisores, com base nos critérios de inclusão e exclusão previamente definidos.

Os estudos considerados potencialmente elegíveis foram posteriormente recuperados na íntegra e avaliados em texto completo quanto ao cumprimento dos critérios de elegibilidade. As divergências entre os revisores foram resolvidas por consenso, garantindo a robustez do processo de seleção. Sempre que o texto integral não estava disponível, foi contactado o autor correspondente e/ou solicitada a obtenção do artigo à biblioteca de uma universidade.

A análise descritiva e a síntese dos dados foram realizadas de forma independente e cega por dois ou mais revisores, utilizando quadros de extração de dados desenvolvidos pela equipa de investigação. Esses quadros incluíram dimensões de caracterização das publicações e dos estudos selecionados, bem como a categorização das intervenções segundo as situações de vulnerabilidade associadas à parentalidade, o contexto de aplicação, as tecnologias digitais utilizadas e as técnicas de mudança comportamental identificadas. Essa categorização está em consonância com os objetivos e a questão da revisão. Após a revisão mútua dos registos, as divergências foram clarificadas com o apoio de um terceiro revisor, culminando na apresentação dos resultados consensualizados.

## RESULTADOS

A pesquisa nas bases de dados permitiu identificar um total de 984 registos. Do processo de seleção e elegibilidade, foram incluídas dez publicações científicas nesta revisão. Os resultados obtidos com o processo de triagem foram apresentados de acordo com o fluxograma PRISMA-ScR 2020^(27)^ (Figura 1).

**Figura 1:** Fluxograma do processo de identificação, triagem, elegibilidade e inclusão dos estudos – Lisboa, Portugal, 2025.

Os resultados da presente *scoping review* foram apresentados de forma descritiva e organizados em quadros sinópticos, de modo a permitir uma visão abrangente das intervenções educativas digitais identificadas, das situações de vulnerabilidade associadas à parentalidade, dos contextos de aplicação, dos recursos tecnológicos utilizados e das técnicas de mudança comportamental mobilizadas. Os quadros seguintes sintetizam as principais características e informações dos estudos incluídos.

O Quadro 1 apresenta a caracterização geral dos estudos incluídos, nomeadamente o ano de publicação, o título, o país de origem, o tipo de estudo, o seu objetivo, os participantes e os moderadores da intervenção.

**Quadro 1:** Caracterização geral das publicações incluídas na revisão – Lisboa, Portugal, 2025.

Autores | Ano | Título do artigo	País	Tipo de estudo	Objetivo	Participantes	Moderadores da intervenção
Anne H. Salonen, Karen F. Pridham, Roger L. Brown, Marja Kaunonen (2014)^(28)^ Impact of an internet-based intervention on Finnish mothers’ perceptions of parenting satisfaction, infant centrality and depressive symptoms during the postpartum year	Finlândia	Quase-experimental e longitudinal com grupo de controlo	Avaliar de que forma uma intervenção baseada na internet influencia a satisfação parental das mães, a centralidade do bebé e os sintomas depressivos durante o primeiro ano pós-parto.	760 mães no período perinatal	Enfermeiros
Michael G. Sawyer, Christy E. Reece, Kerrie Bowering, Debra Jeffs, Alyssa C. P. Sawyer, Murthy Mittinty, John W. Lynch (2017)^(29)^ Nurse-Moderated Internet-Based Support for New Mothers: Non-Inferiority, Randomized Controlled Trial	Austrália	Ensaio clínico controlado randomizado de não inferioridade	Testar a não inferioridade dos resultados para mães e bebés que receberam uma avaliação de saúde pósnatal realizada em contexto clínico, acompanhada por apoio em grupo moderado por enfermeiro através da internet, em comparação com os resultados das mães e bebés que receberam os cuidados padrão, consistindo em apoio pós-natal domiciliário prestado por um enfermeiro de comunidade.	819 mulheres durante o pós-parto que tiveram alta hospitalar, com filhos entre 1 e 7 meses de idade	Enfermeiros
Deborah E. McCarter, Eugene Demidenko &. Mark T. Hegel (2018)^(30)^ Measuring outcomes of digital technology-assisted nursing postpartum: A randomized controlled trial	Estados Unidos da América	Longitudinal-coorte em três fases: fases 1e 2 foram estudos de viabilidade e aceitabilidade; fase 3 foi um ensaio clínico controlado randomizado	Determinar se o envio de mensagens eletrónicas por parte de enfermeiros durante os primeiros seis meses após o parto é viável, aceitável e eficaz na melhoria do estado de espírito e na redução do stress parental.	Fase 1 – 75 mulheres durante o pós-parto Fase 2 – 55 mulheres durante o pós-parto Fase 3 – 547 mulheres durante o pós-parto	Enfermeiros
J. Banerjee, A. Aloysius, K. Platonos, & A. Deierl (2018)^(31)^ Innovations: Supporting family integrated care	Reino Unido		Analisar formas inovadoras de quebrar a barreira da educação e da comunicação, bem como o acesso dos pais ao berço, através de ideias inovadoras e tecnologias digitais.		Enfermeiros
Cigdem Sari & Naime Altay (2020)^(32)^ Effects of providing nursing care with webbased program on maternal self-efficacy and infant health	Turquia	Ensaio clínico randomizado com desenho pós-teste	Examinar a eficácia de um programa online (baseado no Modelo de Promoção da Saúde de Pender), dirigido a mulheres primíparas, no crescimento e desenvolvimento do lactente, na saúde dos lactentes e no nível de autoeficácia das mulheres.	71 mulheres primíparas e os seus bebés recrutadas de três clínicas de cuidados de saúde primários	Enfermeiros
Annica Sjöström Strand, Björn Johnsson, Momota Hena, Boris Magnusson, Inger Kristensson Hallström (2022)^(33)^ Developing eHealth in neonatal care to enhance parents’ self-management	Suécia	Qualitativo	Desenvolver uma aplicação eHealth que facilite a transição do hospital para casa de pais com bebés prematuros, que substitua ou complemente visitas presenciais aos serviços de cuidados de saúde e que ofereça um canal de comunicação direto, seguro e eficaz com os profissionais de saúde, promovendo o autocuidado e a segurança familiar.	Três famílias testaram o protótipo ainda durante o internamento hospitalar; na fase seguinte, três famílias testaram o protótipo em ambiente hospitalar; e cinco famílias com lactentes que nasceram com idade gestacional entre as 24–34 semanas testaram o mesmo protótipo no seu contexto domiciliário	Enfermeiros
Antonio Di Mauro, Federica Di Mauro, Chiara Greco, Orazio Valerio Giannico, Francesca Maria Grosso, Maria Elisabetta Baldassarre, Manuela Capozza, Federico Schettini, Pasquale Stefanizzi, Nicola Laforgia (2021)^(34)^ *In-hospital and web-based intervention to counteract vaccine hesitancy in very preterm infants’ families: a NICU experience*	Itália	Observacional quantitativo	Avaliar a cobertura e a pontualidade das vacinas do programa nacional de vacinação, em uma coorte de bebés prematuros (< 33 semanas de idade gestacional) aos 24 meses de idade, cujas famílias foram sujeitas à intervenção, tanto no hospital como online, para combater a hesitação vacinal.	Pais de 170 recém-nascidos que nasceram antes das 33 semanas de idade gestacional e estiveram na Unidade de Cuidados Neonatais, com acesso à internet e com perfil no Facebook®	Enfermeiros da área neonatal e médicos neonatologistas
Lisa Militello, Emre Sezgin, Yungui Huang, Simon Lin (2021)^(35)^ *Delivering Perinatal Health Information via a Voice Interactive App (SMILE): Mixed Methods Feasibility Study*	Estados Unidos da América	Viabilidade de métodos mistos	Avaliar a viabilidade do uso da tecnologia de voz para apoiar a saúde perinatal e as práticas de cuidados com o bebé.	19 mulheres grávidas (17 a 36 semanas de gestação)	
Joelle Yan Xin Chua, Mahesh Choolani, Cornelia Yin Ing Chee, Huso Yi, Yiong Huak Chan, Joan Gabrielle Lalor, Yap Seng Chong, Shefaly Shorey (2024)^(36)^ *Parents’ Perceptions of Their Parenting Journeys and a Mobile App Intervention (Parentbot-A Digital Healthcare Assistant): Qualitative Process Evaluation*	Singapura	Qualitativo e descritivo	Explorar as experiências perinatais de pais em Singapura e examinar as experiências do usuário da intervenção baseada em aplicação móvel com um chatbot integrado chamado Parentbot — um assistente de saúde digital (PDA).	118 casais heterossexuais com mais de 21 anos de idade recrutados por meio de amostragem intencional em um hospital terciário em Singapura	
Sahiti Myneni, Angela Ross, Alexandra Zingg, Tavleen Singh, Amy Franklin, Deevakar Rogith, Jerrie Refuerzo (2024)^(37)^ *Digital health technologies for high-risk pregnancy management: three case studies using Digilego framework*	Estados Unidos da América	Métodos mistos	Desenvolver e avaliar três soluções digitais para apoiar mulheres com gravidez de alto risco (diabetes gestacional, hipertensão e depressão perinatal) através do Digilego framework.	Estudo 1: 9.364 mulheres que interagiram em duas aplicações de gravidez: “What to Expect” e “Baby Center”; Estudo 2: dez mulheres grávidas testaram a “MyPregnancyChart”; Estudo 3: 30 mulheres grávidas testaram a “MomMind”	

As dez publicações científicas incluídas nesta scoping review correspondem aos estudos que cumpriram integralmente os critérios de elegibilidade definidos e resultaram do processo de identificação, seleção e inclusão apresentado no fluxograma PRISMA-ScR. Esses estudos foram publicados entre 2013 e 2024, intervalo que corresponde ao período de publicação dos estudos incluídos em oito países distintos, nomeadamente Estados Unidos da América (três estudos)^(30,35,37)^, Suécia (um estudo)^(33)^, Singapura (um estudo)^(36)^, Itália (um estudo)^(34)^, Austrália (um estudo)^(29)^, Turquia (um estudo)^(32)^, Finlândia (um estudo)^(28)^ e Reino Unido (um estudo)^(31)^.

As publicações consideradas foram classificadas individualmente, a partir das quais se obtiveram dois estudos qualitativos^(33,36)^, um estudo observacional quantitativo^(34)^, um estudo de viabilidade de métodos mistos^(35)^, um estudo longitudinal-coorte em três fases^(30) —^ fases 1 e 2 foram estudos de viabilidade e aceitabilidade; fase 3 foi um ensaio clínico controlado randomizado —, um estudo de métodos mistos^(37)^, um estudo quase-experimental longitudinal^(28)^, um ensaio clínico randomizado de não inferioridade^(29)^ e um ensaio clínico randomizado com desenho pós-teste^(32)^.

Após a análise integral dos estudos selecionados, foram identificadas intervenções educativas com recurso a tecnologias digitais promotoras do *empowerment* parental dirigidas a pais em situação de vulnerabilidade. Verificou-se que, das intervenções identificadas, a maioria foi moderada por enfermeiros, sendo que uma dessas foi moderada também por médicos neonatalogistas em parceria com os enfermeiros que exercem funções na Unidade de Cuidados Neonatais (UCN) onde o estudo foi realizado.

A análise dos dados permitiu identificar cinco categorias emergentes: Situações de vulnerabilidade na parentalidade; Contexto de aplicação da intervenção educativa com recurso a tecnologias digitais; Tecnologia digital em uso; Intervenções educativas com recurso a tecnologias digitais; Técnicas de mudança comportamental na intervenção para promoção do *empowerment* parental. Os resultados dessa análise foram apresentados no Quadro 2.

**Quadro 2:** Síntese dos estudos incluídos na revisão da literatura – Lisboa, Portugal, 2025.

Citação | Ano	Situações de vulnerabilidade na parentalidade	Contexto de aplicação da intervenção educativa com recurso a tecnologias digitais	Tecnologia digital em uso	Intervenções educativas com recurso a tecnologias digitais	Técnicas de mudança comportamental na intervenção para promoção do empowerment parental
Salonen et al., 2014 ^(28)^	Transição para a parentalidade (períodos prée pós-natal) – mulheres primíparas e multíparas durante a gravidez e até um ano pós-parto	Contexto domiciliário	Website	A intervenção com recurso ao website foi projetada para fortalecer a satisfação parental e a autoeficácia parental, oferecendo suporte online para parentalidade, amamentação e cuidados infantis. A website consiste em: (a) um banco de dados de informações; (b) um fórum de discussão entre pares online; e (c) um serviço de perguntas/respostas, focando-se na satisfação parental, na centralidade infantil e em sintomas depressivos.	Transmissão de informação e modificação do ambiente [social]
Sawyer et al., 2017^(29)^	Pós-parto e adaptação à parentalidade – mães no período pós-parto (até sete meses pós-parto)	Em domicílio (a partir de 1 mês até 7 meses de idade das crianças)	Website	Através do website, acessível pelo computador ou pelo telemóvel, as mães podem comunicar entre si através de um fórum, colocando questões sobre as suas dificuldades e esclarecendo-se e apoiandose mutuamente. A comunicação entre as mães é moderada por um enfermeiro, que esclarece as mesmas com base na evidência científica.	Transmissão de informação e modificação do ambiente [social]
McCarter et al., 2018^(30)^	Pós-parto e adaptação à parentalidade – mães no período pós-parto (até seis meses pós-parto)	Início no hospitalar e após em domicílio	Mensagens e chamadas telefónicas	Com o envio de mensagens educacionais, é fornecida informação sobre o autocuidado na maternidade e os cuidados com a criança. Além do conteúdo informativo, uma enfermeira experiente na área materno-infantil envia mensagens de inspiração e suporte. Grupo I – quatro mensagens/semana durante seis meses; Grupo II – quatro mensagens/semana, sendo que duas oferecem a possibilidade de responder e solicitar chamada telefónica com enfermeira durante seis meses.	Transmissão de informação e regulação de emoções
Banerjee et al., 2018(31)	Parentalidade em contexto de hospitalização neonatal – pais de recém-nascidos hospitalizados em Unidade de Cuidados Neonatais	Contexto hospitalar Aplicação móvel digital passível de utilização em contexto de domicílio	*Aplicação móvel digital “Integrated Family Delivered Care (IFDC)” ^**^Headphones canceladores de ruído ^**^Impressão digital para acesso à Unidade de Cuidados Neonatais ^***^Sistema de mensagens de vídeo	Por meio de aplicação móvel digital, os pais conseguem ter acesso a inúmeros conteúdos de índole educacional e podem ainda partilhar a jornada da parentalidade com recém-nascidos pré-termo. A utilização de headphones canceladores de ruído garantem privacidade dos recém-nascidos e das famílias na Unidade de Cuidados Neonatais aquando das passagens de turno, reduzindo a interrupção da vinculação com os seus recém-nascido durante esses períodos. Através do leitor biométrico por impressão digital, os pais podem ter acesso contínuo à Unidade de Cuidados Neonatais. Com a partilha de mensagens de vídeo entre os profissionais de saúde e os pais, são fornecidas atualizações frequentes sobre o estado dos recém-nascidos admitidos na Unidade de Cuidados Neonatais, facilitando a vinculação quando não é possível a presença parental.	*Transmissão de informação, *demonstração do comportamento, ^**^modificação do ambiente [físico], regulação de emoções e ***regulação de emoções
Sari & Altay, 2020^(32)^	Transição para a parentalidade (períodos prée pós-natal) – fase prénatal: mulheres grávidas com 32–37 semanas de gestação; fase pós-natal: até aos três meses	O website é introduzido na fase pré-natal no contexto de cuidados de saúde primários, utilizado durante a gravidez e no período pós-natal no domicílio	Website	Por meio do website, que se encontra dividido em quatro tópicos, de acordo com o Modelo de Promoção da Saúde de Pender – “My Baby’s Nutrition”, “My Baby’s Bath and Care”, “My Baby’s Sleep and Safety” e “Communication with My Baby” -, foram fornecidos conteúdos informativos sobre os mesmos tópicos. Além do supracitado, através deste website, os pais têm acesso a vídeos compartilhados com informação e demonstrações sobre os diversos tópicos, mensagens motivacionais e contacto com profissionais de saúde.	Transmissão de informação, regulação de emoções e demonstração do comportamento
Strand et al., 2022^(33)^	Parentalidade em contexto de hospitalização neonatal – pais de bebés nascidos prematuros (com 24 a 34 semanas de idade gestacional), em transição para a alta hospitalar	A aplicação da intervenção digital em desenvolvimento foi realizada tanto em contexto hospitalar quanto em contexto domiciliário	Aplicação móvel digital de eHealth adaptada a tablets Android	Através da aplicação móvel digital, os enfermeiros da Unidade de Cuidados Neonatais conseguem se comunicar com os pais através de mensagens de texto, fotografias e videochamadas. Adicionalmente, através da aplicação móvel digital, é possível registar e monitorizar dados clínicos do bebé (peso, níveis de oxigenação, ingestão alimentar), permitindo que os enfermeiros concretizem um acompanhamento à distância. Ao utilizar essa aplicação, os profissionais de saúde conseguem ainda fazer a observação e avaliação do bebé, através da realização das videochamadas.	Transmissão de informação e regulação de emoções
Di Mauro et al., 2021^(34)^	Parentalidade em contexto de hospitalização neonatal – pais de recém-nascido pré-termo com até 2 anos de idade	Divulgação em consultas de cuidados de saúde primários aos 2, 4, 6 e 12 meses de vida e uso em domicílio	Página na rede social “Facebook®”	Através da página de Facebook®, os pais conseguem ter acesso a um conteúdo claro e simples sobre os riscos/benefícios das vacinas, as doenças prevenidas pela vacinação e as idades recomendadas para as vacinas segundo o programa de vacinação italiano.	Transmissão de informação
Militello et al., 2021^(35)^	Transição para a parentalidade (períodos prée pós-natal) – mulheres e famílias em fase perinatal entre 17 e 36 semanas de gestação	Contexto domiciliário	Aplicação móvel digital interativa de voz chamada “Self-Management Intervention–Life Essentials (SMILE)”	Através da aplicação móvel digital, é disponibilizada informação por meio de podcasts sequencias com durações de três a oito minutos sobre as necessidades do bebé e cuidados, cuidados pósparto e depressão pós-parto, alimentação do bebé/amamentação, e estratégias para gerir a relação conjugal, enfocando na educação perinatal. Ao fim de cada mini-podcast, os pais transmitiam feedback sobre cada um através de gravações de áudio. Adicionalmente, foram disponibilizadas ligações para documentos fidedignos e uma lista de contactos úteis para acesso a recursos profissionais.	Transmissão de informação
Chua et al., 2024^(36)^	Transição para a parentalidade (períodos prée pós-natal) – pais no período perinatal e pósnatal (desde 24 semanas de gestação até um mês após o parto)	Intervenção apresentada em meio hospitalar com aplicação em domicílio	Aplicação móvel digital com um chatbot denominada “Parentbot” — um assistente digital de saúde (PDA)	Por meio da aplicação móvel digital, foi fornecido aos pais suporte informativo, psicológico e social durante a gravidez (> 24 semanas de gestação) até o primeiro mês após o parto. O suporte supracitado é mediado por: – Materiais educativos; – Fórum de discussão/apoio; – Atividades para regulação emocional; – Chatbot integrado.	Transmissão de informação, regulação de emoções e modificação do ambiente [social]
Myneni et al., 2024(37)	Fase perinatal em mulheres com gravidez de alto risco (diabetes gestacional, hipertensão e depressão periparto)	Utilizada durante a gravidez em contexto de ambulatório	Duas aplicações digitais: *“MyPregnancyChart” e ^**^“MomMind”	Através da aplicação MyPregnancyChart, as mulheres grávidas podem abordar várias temáticas em um fórum de discussão online e registar dados clínicos, permitindo que o sistema sinalize quando aconselhável a avaliação médica, atendendo, assim, à vigilância, à manutenção e/ou à mudança de comportamento. Através da aplicação MomMind, as mães têm acesso a um repositório categorizado por tema que auxilia a identificação de sintomas de depressão, ensina estratégias para lidar com o stress, tristeza ou ansiedade, e reconhece e normaliza as dificuldades emocionais sentinas no periparto.	*Modificação do ambiente [social], ^**^transmissão de informação e ^**^regulação de emoções

Com vistas a aprofundar sobre a clareza analítica desta *scoping review*, as intervenções educativas com recurso a tecnologias digitais foram sistematizadas através de quatro técnicas de mudança comportamental – transmissão de informação, demonstração do comportamento, modificação do ambiente e regulação de emoções -, permitindo evidenciar a forma como os elementos contribuem para o *empowerment* parental em situações de vulnerabilidade, conforme representado na Figura 2.

**Figura 2:** Técnicas de mudança comportamental mobilizadas nas intervenções educativas digitais e sua contribuição para o *empowerment* parental em situações de vulnerabilidade – Lisboa, Portugal, 2025.

## DISCUSSÃO

### Situações de Vulnerabilidade na Parentalidade

No âmbito desta *scoping review*, a vulnerabilidade na parentalidade é compreendida como uma condição dinâmica e multidimensional, resultante da interação entre fatores individuais, clínicos, psicossociais e contextuais, que podem comprometer a capacidade dos pais para exercerem a sua parentalidade de forma informada, segura e confiante. Essas situações incluem pais e famílias que, em determinados momentos do ciclo vital ou em contextos específicos de cuidados de saúde, enfrentam exigências acrescidas, incerteza, stress emocional ou limitações no acesso a recursos e apoio profissional.

Os estudos incluídos evidenciam que a vulnerabilidade na parentalidade emerge, sobretudo, em períodos de transição e adaptação, bem como em contextos clínicos complexos, nos quais os pais são confrontados com novas responsabilidades, decisões críticas e necessidades de aprendizagem intensiva.

As situações de vulnerabilidade descritas nos estudos incluídos incidem sobre quatro eixos: (1) transição para a parentalidade (período pré e pós-natal); (2) pós-parto e adaptação à parentalidade; (3) gravidez de alto risco e vulnerabilidade clínica materna; e (4) parentalidade em contexto de hospitalização neonatal. Globalmente, as populações abrangem o período gestacional, o período pós-parto e o período neonatal, estendendo-se à primeira infância, até aos 36 meses, fase reconhecida como crítica para o desenvolvimento infantil e para a consolidação das competências parentais.

No que diz respeito à transição para a parentalidade, com enfoque nos períodos pré e pós-natal, quatro estudos incidiram sobre o período gestacional: mulheres entre 17–36 semanas^(35)^; pais a partir de 24 semanas^(36)^; grávidas entre 32–37 semanas^(32,37)^. Nesses contextos, a vulnerabilidade pode se associar à antecipação do exercício da parentalidade, à necessidade de aquisição de conhecimentos e competências e à gestão de expectativas e emoções diante da parentalidade iminente.

Relativamente ao pós-parto e à adaptação à parentalidade, cinco estudos incluíram o período pós-natal^(28,29,30,31,32,36)^, com acompanhamento até um mês^(36)^, seis meses^(30)^ e um ano^(28)^. Dois estudos centraram-se especificamente em mães até três meses^(29)^e sete meses^(32)^. Nesses casos, a vulnerabilidade pode emergir da sobrecarga física e emocional, das dificuldades na adaptação ao novo papel e da necessidade de apoio continuado na gestão dos cuidados ao RN.

Apenas um estudo teve enfoque na gravidez de alto risco, sendo especificamente direcionado a mulheres com hipertensão/diabetes gestacional e depressão periparto^(37)^.

Em relação à parentalidade em contexto de hospitalização neonatal, foram identificados três estudos, que foram desenvolvidos com: pais de RNs internados em UCN^(31)^; pais de bebés prematuros (24–34 semanas) em transição para a alta^(33)^; e pais de RN pré-termo até aos 2 anos^(34)^. Em conjunto, esses estudos parecem evidenciar uma vulnerabilidade prolongada associada à prematuridade, à hospitalização e à continuidade de cuidados após a alta caracterizada pela ansiedade, pela necessidade de informação especializada e pelo apoio na aquisição de competências parentais em contextos altamente tecnificados.

### Contexto de Aplicação da Intervenção Educativa com Recurso a Tecnologias Digitais

Em consonância com o objetivo desta *scoping review* de mapear os contextos de aplicação das intervenções educativas digitais promotoras do *empowerment* parental, os resultados revelam que essas intervenções são desenvolvidas em diferentes contextos, refletindo a diversidade de necessidades das famílias e a flexibilidade dessas ferramentas em se adaptarem a diferentes ambientes.

Nos estudos incluídos, são descritas intervenções com recurso a tecnologias digitais apresentadas em contexto de cuidados de saúde e utilizadas pela população-alvo em contexto hospitalar e/ou domiciliário. Por exemplo, três estudos desenvolvidos descrevem intervenções com recurso a tecnologias digitais que, embora iniciadas em meio hospitalar, são também projetadas para uso em domicílio, promovendo a continuidade dos cuidados para além da alta^(30,31,33,36)^. Um desses estudos descreve não só a possibilidade de utilização domiciliária da aplicação IFDC, como também soluções exclusivamente hospitalares, como auscultadores com cancelamento de ruído e acesso biométrico à UCN^(31)^.

Outros estudos descrevem intervenções educativas concebidas para uso em domicílio, ainda que apresentadas em contexto clínico. Exemplos incluem intervenções introduzidas por equipas hospitalares^(30,36)^ em cuidados de saúde primários^(32,34)^ ou em ambulatório/consultas^(37)^ para posterior utilização pelos pais no domicílio. Esses resultados deixam transparecer o potencial das tecnologias digitais enquanto eventuais mediadores da transição entre contextos de cuidado. Em particular, em um deles, os autores divulgaram a tecnologia em uso durante as consultas realizadas aos dois, quatro, seis e 12 meses de vida da criança^(34)^.

### Intervenções Educativas com Recurso a Tecnologias Digitais

Os estudos incluídos abordaram diversas tecnologias digitais como recurso para promover o *empowerment* parental em contextos de vulnerabilidade. As aplicações destacaram-se como o recurso mais frequente, presentes em cinco artigos^(29,30,31,33,35,36,37)^, com funcionalidades que variaram entre *eHealth* ^(33)^, assistente de voz^(35)^, *chatbot* de apoio parental^(36)^, suporte a famílias em UCN^(31)^ e aplicações para informação e suporte emocional às mães^(37)^.

A segunda tecnologia digital mais recorrentemente mobilizada nos estudos incluídos foram plataformas digitais e *websites* ^(28,29,32,34)^. Destaca-se uma página na rede social Facebook^®(34)^ e *websites* educativos de navegação intuitiva^(28,29,32)^. Essa diversidade de recursos tecnológicos evidencia diferentes estratégias de acesso à informação e de apoio parental, em função do contexto e das características da população-alvo.

Alguns estudos recorreram ainda a outras tecnologias digitais, como o envio de mensagens de texto, chamadas telefónicas^(30)^ ou a utilização de um sistema de mensagens de vídeo para facilitar a comunicação entre pais e equipa de saúde^(31)^. Duas tecnologias digitais complementares identificadas durante esse processo foram o acesso livre à UCN pelos pais por impressão digital e a utilização de *headphones* com cancelamento de ruído^(31)^. Apesar da diversidade de recursos tecnológicos, os estudos convergem na valorização da acessibilidade, interatividade e personalização como elementos-chave para o sucesso das intervenções digitais de apoio à parentalidade em contextos de vulnerabilidade.

### Intervenções Educativas com Recurso a Tecnologias Digitais na Promoção do Empowerment Parental em Situações de Vulnerabilidade

As intervenções educativas com recurso a tecnologias digitais promotoras do *empowerment* parental em situações de vulnerabilidade identificadas nesta revisão foram classificadas em quatro categorias, correspondentes às técnicas de mudança comportamental aplicadas: (i) transmissão de informação; (ii) demonstração do comportamento; (iii) modificação do ambiente; e (iv) regulação de emoções^(9)^.

A maioria das intervenções agrupa-se na transmissão de informação, isto é, centradas na promoção do conhecimento parental sobre conceitos e temáticas essenciais a desenvolver e adquirir na parentalidade^(28,29,30,31 32,33 34,35,36,37)^. Dessas intervenções, apenas duas apresentam a transmissão de informação como única e exclusiva forma para promover o *empowerment* parental^(34,35)^.

Quatro dos estudos identificados combinam transmissão de informação com regulação de emoções, recorrendo a estratégias desenhadas para reduzir a ansiedade/medo e fortalecer a autoeficácia^(30,32,36,37)^. Em outro estudo, uma intervenção predominantemente informativa pareceu contribuir, de forma indireta e consequencial, para a regulação emocional dos pais, com eles reportando maior autoeficácia, segurança e confiança no desempenho parental^(33)^. Esses achados evidenciam a interrelação entre componentes informativos e emocionais no processo de *empowerment* parental.

Estudo descreve uma intervenção digital que promove o *empowerment* parental exclusivamente através da regulação das emoções, utilizando uma plataforma *online* para envio de mensagens informativas e de reforço positivo, além de conteúdo multimédia sobre o estado e desenvolvimento do RN, permitindo acompanhamento dos marcos significativos à distância^(31)^.

A demonstração do comportamento surge em duas intervenções – um *website* ^(32)^ e a aplicação “*Integrated Family Delivered Care*”^(31)^ -, que disponibilizam vídeos demonstrativos dos cuidados, além de conteúdos informativos e componentes de regulação emocional.

Destaca-se que seis das 14 intervenções são promotoras do *empowerment* parental através da modificação do ambiente: quatro a nível do ambiente social^(28,29,36,37)^ – através da criação de fóruns de discussão *online* entre pais) – e duas no ambiente físico – através do uso de *headphones* ^(31)^ com cancelamento de ruído para permanência na UCN e acesso por impressão digital^(31)^ à unidade. Essas estratégias contribuíram também para a regulação de emoções (redução da ansiedade de separação e da angústia associada a dificuldades de acesso).

Em síntese, os resultados parecem evidenciar que, enquanto algumas intervenções operam por um único componente comportamental, outras combinam múltiplas categorias, frequentemente interrelacionadas e com efeitos consequenciais sobre o *empowerment* parental, respondendo, de forma diferenciada, às situações de vulnerabilidade identificadas.

## CONCLUSÃO

Esta *scoping review* permitiu mapear as intervenções educativas com recurso a tecnologias digitais promotoras do *empowerment* parental em situações de vulnerabilidade, evidenciando a diversidade de contextos, recursos tecnológicos e técnicas de mudança comportamental mobilizadas ao longo da transição para e no exercício da parentalidade.

Os resultados sugerem que as tecnologias digitais constituem recursos particularmente relevantes em contextos de vulnerabilidade, ao permitirem responder, de forma flexível, contínua e personalizada, às necessidades informativas e emocionais dos pais, especialmente nos períodos prée pós-natal e em contextos de hospitalização neonatal. Nesse sentido, o *empowerment* parental emerge não apenas como um resultado esperado, mas como um processo dinâmico, sustentado pela combinação de informações baseadas em evidências, apoio emocional e modificação do ambiente de cuidados.

As intervenções educativas digitais identificadas foram maioritariamente mediadas por enfermeiros, reforçando a intervenção desses profissionais como agentes centrais na promoção da parentalidade, da literacia em saúde e da autonomia parental, em consonância com uma abordagem centrada na família. A integração dessas intervenções parece poder ampliar o alcance da intervenção educativa para além do contacto presencial, favorecendo a continuidade dos cuidados e o fortalecimento da relação e comunicação entre pais e profissionais de saúde.

O principal contributo desta revisão reside na organização das intervenções educativas digitais segundo técnicas de mudança comportamental, permitindo uma leitura mais explicativa sobre os mecanismos através dos quais essas intervenções podem promover o *empowerment* parental em situações de vulnerabilidade.

Apesar do potencial identificado, persistem lacunas relevantes na literatura, nomeadamente a escassez de estudos com desenho experimental ou quase-experimental, a limitada avaliação do impacto a médio e longo prazo das intervenções e a reduzida exploração da perspetiva dos profissionais de saúde envolvidos na sua implementação. Sugere-se que futuras investigações privilegiem o desenvolvimento e a avaliação de intervenções digitais co-desenhadas com pais e profissionais, integrando múltiplas técnicas de mudança comportamental e avaliando resultados centrados no *empowerment* parental e nos processos de transição na e para a parentalidade.

Esta revisão não incluiu a avaliação da qualidade metodológica dos estudos, em conformidade com a metodologia JBI para *scoping reviews*, o que limita as inferências sobre robustez dos efeitos. Embora tenha sido realizada uma pesquisa abrangente em múltiplas bases de dados, a ausência de busca sistemática em bases internacionais de teses e dissertações e a limitação da pesquisa no *Google Scholar* às primeiras dez páginas de resultados poderão ter condicionado a identificação de toda a evidência potencialmente relevante. Esses aspetos reforçam a necessidade de estudos primários metodologicamente rigorosos e de futuras revisões sistemáticas que aprofundem a avaliação da efetividade das intervenções educativas digitais promotoras do *empowerment* parental.

## Data Availability

Todo o conjunto de dados que dá suporte aos resultados deste estudo foi publicado no próprio artigo.

# Translation: Intervenciones educativas que utilizan tecnologías digitales para promover el empowerment parental en situaciones de vulnerabilidad: una revisión de alcance

**DOI:** 10.1590/1980-220X-REEUSP-2025-0464es

**Authors:** Joana Rita Guarda da Venda Rodrigues, Inês Miranda Teixeira, Joana Margarida Marques de Sousa da Piedade, Joana Santos Silva, Mariana Lopes Gonçalves, Bruno Ricardo Ferreira dos Santos Gomes, Olga Maria Martins de Sousa Valentim

**Affiliations:** 1 Universidade de Lisboa, Escola Superior de Enfermagem, Centro de Investigação Inovação e Desenvolvimento em Enfermagem de Lisboa, Lisboa, Portugal.; 2 Unidade Local de Saúde de Lisboa Ocidental, Hospital de São Francisco Xavier, Unidade de Neonatologia, Lisboa, Portugal.; 3 Unidade Local de Saúde do Algarve, Hospital de Faro, Serviço de Medicina Intensiva Neonatal e Pediátrica, Faro, Portugal.; 4 Unidade Local de Saúde de São José, Hospital Dona Estefânia, Unidade de Cuidados Intensivos Neonatais, Lisboa, Portugal.; 5 Unidade Local de Saúde de São José, Maternidade Dr. Alfredo da Costa, Serviço de Neonatologia, Lisboa, Portugal.; 6 Unidade Local de Saúde de São José, Hospital Dona Estefânia, Serviço de Infecciologia, Lisboa, Portugal.

**Keywords:** Responsabilidad Parental, Vulnerabilidad ante Desastres, Tecnología Digital, Empoderamiento, Educación en Salud

## Abstract

**Objetivo:**

Mapear las intervenciones educativas que utilizan tecnología digital para promover el empoderamiento parental en situaciones vulnerables.

**Método:**

Revisión de alcance según la metodología JBI. Se realizó una búsqueda en las bases de datos CINAHL, MEDLINE, Psychology and Behavioral Science Collection, MedicLatina, Scopus, Cochrane Central Register of Controlled Trials, Cochrane Database of Systematic Reviews, Cochrane Clinical Answers y Web of Science, así como en el portal Repositório Científico de Acesso Aberto de Portugal y el motor de búsqueda Google Scholar. Los criterios de elegibilidad incluyeron padres y familias en situaciones vulnerables, intervenciones educativas digitales que promueven el empoderamiento parental y todos los contextos de intervención.

**Resultados:**

Se identificaron 984 registros y se incluyeron diez estudios. La evidencia se distribuyó en cuatro ejes de vulnerabilidad, abarcando participantes desde el periodo gestacional hasta la primera infancia. Las intervenciones se llevaron a cabo en entornos hospitalarios y/o domiciliarios, principalmente mediante aplicaciones móviles, seguidas de plataformas/sitios web, con uso ocasional de redes sociales, mensajería y video. Predominó la transmisión de información, frecuentemente combinada con la regulación emocional. Se identificaron intervenciones menores que implicaban demostración de comportamiento y modificación del entorno.

**Conclusión:**

Las intervenciones educativas digitales son prometedoras y complementan las intervenciones presenciales, fortaleciendo la relación y la comunicación entre padres y profesionales y permitiendo una atención más accesible, personalizada y continua.

## INTRODUCCIÓN

La parentalidad constituye un foco de atención en la práctica de la atención de enfermería, y se describe formalmente como el reconocimiento y la adopción de las responsabilidades inherentes a ser madre y/o padre. Esta orientación se traduce en comportamientos destinados a facilitar la integración del recién nacido (RN) en la unidad familiar y optimizar su crecimiento y desarrollo^(1)^. Esto puede implicar etapas progresivas de vinculación, adaptación, construcción de la identidad parental y aceptación del nuevo ser, resultado de un proceso de toma de conciencia de nuevas responsabilidades, adquisición de conocimientos y competencias, que surge como una situación de vulnerabilidad^(2,3,4)^.

La vulnerabilidad, aunque puede presentar variaciones según las diferentes dimensiones que le están asociadas, se entiende aquí como la susceptibilidad y el riesgo de sufrir daños a partir de procesos multidimensionales, políticos, económicos, culturales, psicológicos o biomédicos^(5)^, constituyendo un fenómeno dinámico y procesual^(6)^. No obstante, la vulnerabilidad asociada a la parentalidad puede atenuarse mediante intervenciones de apoyo emocional, social y educativo iniciadas por profesionales de la salud y centradas en las familias que promueven su *empowerment* ^(6)^.

En este contexto, el *empowerment* parental se entiende como un proceso a través del cual los padres adquieren un mayor control sobre las decisiones y acciones que influyen en la vida y la salud de sus hijos, tomando mayor conciencia de sus capacidades, fortaleciendo la autoconfianza, adquiriendo conocimientos y desarrollando competencias que les permiten ejercer un papel activo en la promoción del desarrollo infantil y su bienestar^(7)^, en particular en las dimensiones cognitiva, afectiva y conductual^(8)^. En este ámbito, la intervención del enfermero es particularmente relevante en el proceso de *empowerment* parental, que tiene como objetivo promover un entorno familiar saludable y favorable al desarrollo infantil. Este proceso se ve facilitado por intervenciones educativas que valorizan la parentalidad, promueven relaciones colaborativas y movilizan recursos comunitarios^(8)^, y puede consolidarse aún más mediante técnicas de cambio conductual, entre las que se destacan: (i) la transmisión de información, proporcionando conocimientos sobre las consecuencias para la salud de los comportamientos que se deben modificar; (ii) la demostración del comportamiento, recurriendo a la simulación o a recursos visuales; (iii) la modificación del entorno físico y social, favoreciendo la adopción del comportamiento deseado; (iv) la regulación emocional, reduciendo las emociones negativas para facilitar la ejecución del comportamiento deseado^(9)^.

En un período marcado por la rápida evolución, las tecnologías digitales – entendidas como un conjunto de recursos basados en tecnologías de la información y la comunicación, que incluyen plataformas en línea, aplicaciones móviles, redes sociales y dispositivos inteligentes – han transformado profundamente las formas de acceso, producción y intercambio de información en salud^(10,11)^. El uso de estas tecnologías digitales interactivas ha permitido una colaboración más estrecha entre los profesionales de la salud y los usuarios, favoreciendo la adaptación de las intervenciones a las necesidades específicas de las personas y las familias en situación de vulnerabilidad^(10)^. Así, los entornos de salud se han ido reconfigurando mediante intervenciones educativas respaldadas por la tecnología digital, que redefinen la forma en que se concibe e implementa la educación en salud, permitiendo un mayor alcance, una mayor personalización y una mayor interactividad^(11)^. Aunque las tecnologías digitales no sustituyen a las prácticas tradicionales, funcionan como recursos complementarios que promueven la relación y la comunicación entre padres y profesionales^(12)^, contribuyendo al fortalecimiento del conocimiento y los cuidados parentales^(13)^. Sin embargo, a pesar de su reconocido potencial para el *empowerment* parental y la promoción del autocuidado, la integración sistemática de estas tecnologías digitales en salud en los servicios dirigidos a familias en situación de vulnerabilidad, como en la transición a la parentalidad, sigue siendo limitada^(14)^.

Se llevó a cabo una búsqueda preliminar, en la que se identificaron diez revisiones. Entre ellas, se encuentran cuatro revisiones sistemáticas^(15,16,17,18)^, cuatro revisiones de alcance^(14,19,20,21)^, una revisión integrativa^(13)^ y una revisión narrativa^(22)^. En general, las revisiones identificadas no explicitan la contribución específica de las intervenciones digitales al *empowerment* parental, privilegiando la evaluación de la eficacia global de las intervenciones en contextos, grupos de edad y áreas temáticas muy específicos (a saber, embarazo en adolescentes^(15)^, embarazo y período posnatal^(14)^, los periodos prenatal^(18)^, perinatal^(21)^ o neonatal^(16,17,18,19,20,21,22)^, la lactancia materna^(17)^, los recién nacidos prematuros^(13)^ y las familias con niños con necesidades especiales de atención médica^(19)^). Por lo tanto, dada la diversidad de las intervenciones educativas que utilizan tecnologías digitales dirigidas a la parentalidad, así como la heterogeneidad de las poblaciones, los contextos y los recursos tecnológicos identificados en la literatura, la realización de una revisión de alcance constituyó el diseño metodológico más adecuado, ya que permite mapear el alcance, la variedad y la naturaleza de la evidencia existente, así como explorar, de manera exhaustiva, las intervenciones educativas digitales que promueven el *empowerment* parental en situaciones de vulnerabilidad, contribuyendo a la identificación de lagunas de conocimiento y a la orientación de futuras investigaciones.

La presente revisión se distingue, por lo tanto, de las revisiones existentes al centrarse en el mapeo de intervenciones educativas que utilizan tecnologías digitales para promover el *empowerment* parental en situaciones de vulnerabilidad. Presenta un carácter innovador por la organización de las intervenciones identificadas según técnicas de cambio conductual, a saber: transmisión de información, demostración del comportamiento, modificación del entorno y regulación de las emociones. Además, reviste especial relevancia al abarcar de manera transversal la parentalidad desde la gestación hasta la primera infancia (≤36 meses), un período crítico para el desarrollo infantil y, por lo tanto, de especial vulnerabilidad para el neurodesarrollo^(23)^.

Al integrar la dimensión de la vulnerabilidad inherente a la parentalidad y al describir las técnicas de cambio conductual utilizadas en las intervenciones educativas digitales, esta revisión llena un vacío en la literatura al ofrecer una lectura más explicativa de cómo dichas intervenciones pueden promover el *empowerment* parental, contribuyendo a orientar prácticas educativas digitales ajustadas a las necesidades de las familias en transición hacia y en la parentalidad.

En este sentido, se llevó a cabo la presente revisión de alcance, cuyo objetivo general es mapear las intervenciones educativas que utilizan tecnologías digitales para promover el *empowerment* parental en situaciones de vulnerabilidad. Los objetivos específicos fueron: (a) identificar las situaciones de vulnerabilidad asociadas a la parentalidad abordadas en los estudios incluidos; (b) identificar los contextos de aplicación de las intervenciones educativas digitales y los recursos tecnológicos movilizados; (c) identificar las técnicas de cambio conductual utilizadas en las intervenciones educativas digitales que promueven el *empowerment* parental.

Teniendo en cuenta estos objetivos, se pretende responder a la pregunta de la revisión “¿Cuáles son las intervenciones que utilizan tecnologías digitales para promover el *empowerment* de la parentalidad en situaciones de vulnerabilidad?” y a las subpreguntas “¿Cuáles son las situaciones de vulnerabilidad asociadas a la parentalidad abordadas en los estudios incluidos?”, “¿En qué contextos se aplican las intervenciones educativas que utilizan tecnologías digitales?”, “¿Qué tipos de recursos tecnológicos se utilizan en las intervenciones educativas identificadas?” y “¿Cuáles son las técnicas de cambio conductual utilizadas en las intervenciones educativas con recurso a tecnologías digitales que promueven el *empowerment* parental?”.

## MÉTODO

La presente revisión de alcance se llevó a cabo de acuerdo con la metodología JBI^(24)^. La redacción de su implementación siguió las directrices de consenso de la *lista de verificación Preferred Reporting Items for Systematic reviews and Metanalyses extension for Scoping Reviews* (PRISMA-ScR)^(25,26)^. El proceso de selección de estudios se presentó de acuerdo con el diagrama de flujo PRISMA-ScR 2020^(27)^. El protocolo de esta revisión de alcance fue desarrollado por los autores y registrado en el Open Science Framework (https://osf.io/sr6tw/overview).

### Criterios de Elegibilidad

Tras delimitar la problemática y para llevar a cabo la investigación, se definieron los criterios de elegibilidad basados en la Población, el Concepto y el Contexto (PCC). La población de este estudio incluye publicaciones científicas cuya población (P) son los padres y las familias en situación de vulnerabilidad, desde el período perinatal hasta la primera infancia (≤ 36 meses). En cuanto al concepto, se buscó incluir publicaciones científicas que abordaran las intervenciones educativas con el uso de tecnologías digitales que promuevan el *empowerment* parental. En cuanto al contexto (C), se consideraron contextos de intervención sin ninguna limitación, como instituciones de salud, fundaciones o el hogar.

En esta revisión se incluyeron estudios cualitativos, cuantitativos o mixtos (descriptivos-exploratorios, ensayos clínicos aleatorios y no aleatorios, estudios de intervención observacionales) de cualquier nivel de evidencia. Los estudios se consideraron independientemente del año de publicación y del idioma.

Como criterios de exclusión, se consideró el incumplimiento de los criterios de PCC descritos anteriormente, así como los siguientes factores: que la intervención digital y el marco de vulnerabilidad en la parentalidad no fueran claros; padres de niños mayores de 36 meses; las revisiones sistemáticas e integrativas; los proyectos en desarrollo y otros documentos sin resultados empíricos publicados; la inaccesibilidad del artículo tras solicitarlo al autor; y la solicitud de apoyo al Centro de Documentación y Biblioteca.

### Estrategia de Búsqueda e Identificación de los Estudios

Para la identificación de los estudios, se utilizaron bases de datos electrónicas internacionales reconocidas por su amplia cobertura en las áreas de salud, enfermería, psicología y evidencia clínica, con el objetivo de maximizar la sensibilidad de la búsqueda e identificar la literatura publicada relevante para el fenómeno en estudio. Se realizaron búsquedas en las bases de datos de la plataforma *EBSCO host: CINAHL Ultimate*, *MEDLINE Ultimate*, *Psychology and Behavioral Science Collection*, *MedicLatina*, Scopus, *Cochrane Central Register of Controlled Trials, Cochrane Database of Systematic Reviews, Cochrane Clinical Answers* y *Web of Science*. Se llevaron a cabo varias pruebas para refinar la estrategia de búsqueda. También se realizó la búsqueda en el portal Repositorio Científico de Acceso Abierto de Portugal y en el motor de búsqueda *Google Scholar*. Se consideraron las primeras diez páginas, ya que son las que presentan los resultados más relevantes para la búsqueda realizada. La búsqueda se llevó a cabo en marzo de 2025 y se realizó en tres etapas.

Como punto de partida, se realizó una búsqueda bibliográfica en la plataforma *EBSCO*, utilizando bases de datos del área de la salud, como *CINAHL Ultimate* y *Medical MEDLINE Ultimate*, con el fin de identificar las palabras en lenguaje natural y las palabras clave utilizadas con mayor frecuencia en los títulos y resúmenes, así como los términos de indexación. En la segunda etapa, las palabras naturales, las palabras clave y los términos de indexación identificados se combinaron con operadores booleanos y, siempre que fue posible, con el operador asterisco (*) para formar la expresión de búsqueda, que se adaptó a las especificidades de cada base de datos o repositorio. Los términos combinados utilizados incluyeron: “parent*”; “*family*”; “*digital health*”; “*internet-based intervention*”; “*digital technology*”; “*health education*”; “*parental empowerment*”; “*health promotion*”; “*parenting education*”; “neonat*”; y “hospital*”.

En la última etapa, se analizaron las referencias bibliográficas presentes en los registros previamente identificados. La búsqueda incluyó igualmente literatura gris, con el objetivo de identificar estudios potencialmente relevantes; sin embargo, solo se consideraron elegibles para su inclusión los documentos con resultados publicados de conformidad con los criterios de inclusión definidos.

### Extracción, Análisis y Síntesis de los Datos

El análisis y la selección de los registros se realizaron utilizando la plataforma *Qatar Computing Research Institute (Rayyan QCRI)*, a través de la cual se identificaron y eliminaron los registros duplicados.

Tras la eliminación de los duplicados, los registros identificados se sometieron a una única fase de selección de títulos y resúmenes, de acuerdo con la metodología JBI para revisiones de alcance. Esta selección fue realizada de forma independiente por cuatro revisores, basándose en los criterios de inclusión y exclusión previamente definidos.

Los estudios considerados potencialmente elegibles fueron posteriormente recuperados en su totalidad y evaluados en el texto completo en cuanto al cumplimiento de los criterios de elegibilidad. Las discrepancias entre los revisores se resolvieron por consenso, garantizando la solidez del proceso de selección. Siempre que el texto completo no estaba disponible, se contactó al autor correspondiente y/o se solicitó el artículo a la biblioteca de una universidad.

El análisis descriptivo y la síntesis de los datos fueron realizados de forma independiente y ciega por dos o más revisores, utilizando cuadros de extracción de datos desarrollados por el equipo de investigación. Dichas tablas incluían dimensiones de caracterización de las publicaciones y los estudios seleccionados, así como la categorización de las intervenciones según las situaciones de vulnerabilidad asociadas a la parentalidad, el contexto de aplicación, las tecnologías digitales utilizadas y las técnicas de cambio conductual identificadas. Esta categorización está en consonancia con los objetivos y la pregunta de la revisión. Tras la revisión mutua de los registros, las divergencias se aclararon con el apoyo de un tercer revisor, lo que culminó en la presentación de los resultados consensuados.

## RESULTADOS

La búsqueda en las bases de datos permitió identificar un total de 984 registros. Tras el proceso de selección y elegibilidad, se incluyeron diez publicaciones científicas en esta revisión. Los resultados obtenidos con el proceso de selección se presentaron de acuerdo con el diagrama de flujo PRISMA-ScR 2020^(27)^ (Figura 1).

**Figura 1:** Diagrama de flujo del proceso de identificación, selección, elegibilidad e inclusión de los estudios – Lisboa, Portugal, 2025.

Los resultados de la presente revisión de alcance se presentaron de manera descriptiva y se organizaron en cuadros sinópticos, con el fin de ofrecer una visión integral de las intervenciones educativas digitales identificadas, las situaciones de vulnerabilidad asociadas a la parentalidad, los contextos de aplicación, los recursos tecnológicos utilizados y las técnicas de cambio conductual empleadas. Los siguientes cuadros sintetizan las principales características e información de los estudios incluidos.

El Cuadro 1 presenta la caracterización general de los estudios incluidos, en particular el año de publicación, el título, el país de origen, el tipo de estudio, su objetivo, los participantes y los moderadores de la intervención.

**Cuadro 1:** Caracterización general de las publicaciones incluidas en la revisión – Lisboa, Portugal, 2025.

Autores | Año | Título del artículo	País	Tipo de estudio	Objetivo	Participantes	Moderadores de la intervención
Anne H. Salonen, Karen F. Pridham, Roger L. Brown, Marja Kaunonen (2014)^(28)^ *Impact of an internet-based intervention on Finnish mothers’ perceptions of parenting satisfaction, infant centrality and depressive symptoms during the postpartum year*	Finlandia	Casi-experimental y longitudinal con grupo de control	Evaluar cómo una intervención basada en Internet influye en la satisfacción parental de las madres, la centralidad del bebé y los síntomas depresivos durante el primer año posparto.	760 madres en el periodo perinatal	Enfermeros
Michael G. Sawyer, Christy E. Reece, Kerrie Bowering, Debra Jeffs, Alyssa C. P. Sawyer, Murthy Mittinty, John W. Lynch (2017)^(29)^ *Nurse-Moderated Internet-Based Support for New Mothers: NonInferiority, Randomized Controlled Trial*	Australia	Ensayo clínico aleatorizado y controlado de no inferioridad	Evaluar la no inferioridad de los resultados para las madres y los bebés que recibieron una evaluación de salud posparto realizada en un entorno clínico, acompañada de apoyo grupal moderado por enfermeras a través de Internet, en comparación con los resultados de las madres y los bebés que recibieron la atención estándar, consistente en apoyo posparto a domicilio prestado por una enfermera comunitaria.	819 mujeres en el posparto que recibieron el alta hospitalaria, con hijos de entre 1 y 7 meses de edad	Enfermeros
Deborah E. McCarter, Eugene Demidenko &. Mark T. Hegel (2018)^(30^ *Measuring outcomes of digital technology-assisted nursing postpartum: A randomized controlled trial*	Estados Unidos	Longitudinal-cohorte en tres fases: las fases 1 y 2 fueron estudios de viabilidad y aceptabilidad de la ; la fase 3 fue un ensayo clínico controlado aleatorizado	Determinar si el envío de mensajes electrónicos por parte del personal de enfermería durante los primeros seis meses tras el parto es viable, aceptable y eficaz para mejorar el estado de ánimo y reducir el estrés parental.	Fase 1: 75 mujeres durante el posparto Fase 2: 55 mujeres durante el posparto Fase 3: 547 mujeres durante el posparto	Enfermeros
J. Banerjee, A. Aloysius, K. Platonos, & A. Deierl (2018)^(31)^ *Innovations: Supporting family integrated care*	Reino Unido		Analizar formas innovadoras de romper las barreras de la educación y la comunicación, así como el acceso de los padres a la cuna, mediante ideas innovadoras y tecnologías digitales.		Enfermeros
Cigdem Sari & Naime Altay (2020)^(32)^ *Effects of providing nursing care with web-based program on maternal selfefficacy and infant health*	Turquía	Ensayo clínico aleatorizado con diseño posprueba	Evaluar la eficacia de un programa en línea (basado en el Modelo de Promoción de la Salud de Pender), dirigido a mujeres primíparas, en el crecimiento y desarrollo del lactante, en la salud de los lactantes y en el nivel de autoeficacia de las mujeres.	71 mujeres primíparas y sus bebés reclutadas en tres centros de atención primaria	Enfermeros
Annica Sjöström Strand, Björn Johnsson, Momota Hena, Boris Magnusson, Inger Kristensson Hallström (2022)^(33)^ *Developing eHealth in neonatal care to enhance parents’ self-management*	Suecia	Cualitativo	Desarrollar una aplicación *de eSalud* que facilite la transición del hospital al hogar de los padres con bebés prematuros, que sustituya o complemente las visitas presenciales a los servicios de atención sanitaria y que ofrezca un canal de comunicación directo, seguro y eficaz con los profesionales sanitarios, promoviendo el autocuidado y la seguridad familiar.	Tres familias probaron el prototipo durante la hospitalización; en la siguiente fase, tres familias probaron el prototipo en el entorno hospitalario; y cinco familias con bebés nacidos con una edad gestacional de entre 24 y 34 semanas probaron el mismo prototipo en su entorno doméstico	Enfermeros
Antonio Di Mauro, Federica Di Mauro, Chiara Greco, Orazio Valerio Giannico, Francesca Maria Grosso, Maria Elisabetta Baldassarre, Manuela Capozza, Federico Schettini, Pasquale Stefanizzi, Nicola Laforgia (2021)^(34)^ *In-hospital and web-based intervention to counteract vaccine hesitancy in very preterm infants’ families: a NICU experience*	Italia	Observacional cuantitativo	Evaluar la cobertura y la puntualidad de las vacunas del programa nacional de vacunación en una cohorte de bebés prematuros (< 33 semanas de edad gestacional) a los 24 meses de edad, cuyas familias fueron objeto de una intervención, tanto en el hospital como *en línea*, para combatir la reticencia a la vacunación.	Padres de 170 recién nacidos que nacieron antes de las 33 semanas de edad gestacional y estuvieron ingresados en la Unidad de Cuidados Neonatales, con acceso a Internet y con perfil en *Facebook®*	Enfermeros del área neonatal y médicos neonatólogos
Lisa Militello, Emre Sezgin, Yungui Huang, Simon Lin (2021)^(35)^ *Delivering Perinatal Health Information via a Voice Interactive App (SMILE): Mixed Methods Feasibility Study*	Estados Unidos de América	Viabilidad de métodos mixtos	Evaluar la viabilidad del uso de la tecnología de voz para apoyar la salud perinatal y las prácticas de cuidado del bebé.	19 mujeres embarazadas (entre 17 y 36 semanas de gestación)	
Joelle Yan Xin Chua, Mahesh Choolani, Cornelia Yin Ing Chee, Huso Yi, Yiong Huak Chan, Joan Gabrielle Lalor, Yap Seng Chong, Shefaly Shorey (2024)^(36)^ *Parents’ Perceptions of Their Parenting Journeys and a Mobile App Intervention (Parentbot-A Digital Healthcare Assistant): Qualitative Process Evaluation*	Singapur	Cualitativo y descriptivo	Explorar las experiencias perinatales de los padres en Singapur y examinar las experiencias de los usuarios de la intervención basada en una aplicación móvil con un *chatbot* integrado llamado *Parentbot*, un asistente digital de salud (PDA).	118 parejas heterosexuales mayores de 21 años reclutadas mediante muestreo intencional en un hospital terciario e de Singapur	
Sahiti Myneni, Angela Ross, Alexandra Zingg, Tavleen Singh, Amy Franklin, Deevakar Rogith, Jerrie Refuerzo (2024)^(37)^ *Digital health technologies for high-risk pregnancy management: three case studies using Digilego framework*	Estados Unidos de América	Métodos mixtos	Desarrollar y evaluar tres soluciones digitales para apoyar a las mujeres con embarazos de alto riesgo (diabetes gestacional, hipertensión y depresión perinatal) mediante el *marco Digilego*.	Estudio 1: 9364 mujeres que interactuaron en dos aplicaciones de embarazo: *“What to* Expect” y *“Baby* Center”; Estudio 2: diez mujeres embarazadas probaron “MyPregnancyChart”; Estudio 3: 30 mujeres embarazadas probaron *“MomMind”*	

Las diez publicaciones científicas incluidas en esta revisión de alcance corresponden a los estudios que cumplieron íntegramente los criterios de elegibilidad definidos y que resultaron del proceso de identificación, selección e inclusión presentado en el diagrama de flujo PRISMA-ScR. Estos estudios se publicaron entre 2013 y 2024, intervalo que corresponde al periodo de publicación de los estudios incluidos en ocho países distintos, a saber: Estados Unidos de América (tres estudios)^(30,35,37)^, Suecia (un estudio)^(33)^, Singapur (un estudio)^(36)^, Italia (un estudio)^(34)^, Australia (un estudio)^(29)^, Turquía (un estudio)^(32)^, Finlandia (un estudio)^(28)^ y Reino Unido (un estudio)^(31)^.

Las publicaciones consideradas se clasificaron individualmente, a partir de lo cual se obtuvieron dos estudios cualitativos^(33,36)^, un estudio observacional cuantitativo^(34)^, un estudio de viabilidad de métodos mixtos^(35)^, un estudio longitudinal de cohorte en tres fases^(30)^ – las fases 1 y 2 fueron estudios de viabilidad y aceptabilidad; la fase 3 fue un ensayo clínico aleatorizado controlado -, un estudio de métodos mixtos^(37)^, un estudio longitudinal cuasi-experimental^(28)^, un ensayo clínico aleatorizado de no inferioridad de tipo “^“(29)^ y un ensayo clínico aleatorizado con diseño posprueba^(32)^.

Tras el análisis exhaustivo de los estudios seleccionados, se identificaron intervenciones educativas que utilizan tecnologías digitales para promover el *empowerment* parental dirigidas a padres en situación de vulnerabilidad. Se observó que, de las intervenciones identificadas, la mayoría fueron moderadas por enfermeros, y una de ellas fue moderada también por médicos neonatólogos en colaboración con los enfermeros que ejercen en la Unidad de Cuidados Neonatales (UCN) donde se llevó a cabo el estudio.

El análisis de los datos permitió identificar cinco categorías emergentes: Situaciones de vulnerabilidad en la crianza; Contexto de aplicación de la intervención educativa con recurso a tecnologías digitales; Tecnología digital en uso; Intervenciones educativas con recurso a tecnologías digitales; Técnicas de cambio conductual en la intervención para la promoción del *empowerment* parental. Los resultados de este análisis se presentan en la Cuadro 2.

**Cuadro 2:** Síntesis de los estudios incluidos en la revisión bibliográfica - Lisboa, Portugal, 2025.

Cita | Año	Situaciones de vulnerabilidad en la crianza	Contexto de aplicación de la intervención educativa con recurso a tecnologías digitales	Tecnología digital utilizada	Intervenciones educativas con recurso a tecnologías digitales	Técnicas de cambio conductual en la intervención para promover el *empowerment* parental
Salonen *et a/.,* 2014^(28)^	Transición a la parentalidad (periodos prenatal y posnatal): mujeres primíparas y multíparas durante el embarazo y hasta un año después del parto	Entorno doméstico	*Sitio web*	La intervención a través del *sitio web* se diseñó para reforzar la satisfacción parental y la autoeficacia parental, ofreciendo apoyo *en línea* sobre la crianza, la lactancia materna y el cuidado infantil. El *sitio web* consta de: (a) una base de datos de información; (b) un foro de debate entre pares *en línea;* y (c) un servicio de preguntas y respuestas, centrado en la satisfacción parental, la central idad del niño y los síntomas depresivos.	Transmisión de información y modificación del entorno [social]
Sawyer *et al.,* 2017^(29)^	Posparto y adaptación a la crianza: madres en el periodo posparto (hasta siete meses después del parto)	En el hogar (desde 1 mes hasta los 7 meses de edad de los niños)	*Sitio web*	A través del *sitio web,* accesible desde el ordenador o el móvil, las madres pueden comunicarse entre sí mediante un foro, planteando preguntas sobre sus dificultades y aclarándolas, además de apoyarse mutuamente. La comunicación entre las madres está moderada por un enfermero, que les aclara las dudas basándose en la evidencia científica.	Transmisión de información y modificación del entorno [social]
McCarter *et al.,* 2018^(30)^	Posparto y adaptación a la crianza: madres en el periodo posparto (hasta seis meses después del parto)	Inicio en el hospital y posteriormente en el domicilio	Mensajes y llamadas telefónicas	Mediante el envío de mensajes educativos, se proporciona información sobre el autocuidado durante la maternidad y el cuidado del niño. Además del contenido informativo, una enfermera con experiencia en el ámbito materno-infantil envía mensajes de inspiración y apoyo. Grupo 1: cuatro mensajes a la semana durante seis meses; Grupo II: cuatro mensajes a la semana, dos de los cuales ofrecen la posibilidad de responder y solicitar una llamada telefónica con una enfermera durante seis meses.	Transmisión de información y regulación de las emociones
Banerjee *et al.,* 2018^(31)^	Parentalidad en el contexto de la hospitalización neonatal: padres de recién nacidos hospitalizados en la Unidad de Cuidados Neonatales	Contexto hospitalario Aplicación móvil digital que puede utilizarse en el contexto doméstico	* Aplicación móvil digital *"Integrated Family Delivered Care* (IFDC)" ^**^Auriculares con cancelación de ruido ^**^lmpresión digital para acceder a la Unidad de Cuidados Neonatales ^***^Sistema de mensajes de vídeo	A través de la aplicación móvil digital, los padres pueden acceder a numerosos contenidos de carácter educativo y compartir la experiencia de la crianza de los recién nacidos prematuros. El uso de *auriculares* con cancelación de ruido garantiza la privacidad de los recién nacidos y de las familias en la Unidad de Cuidados Neonatales durante los cambios de turno, reduciendo la interrupción del vínculo con sus recién nacidos durante esos periodos. A través del lector biométrico de huellas dactilares, los padres pueden tener acceso continuo a la Unidad de Cuidados Neonatales. Gracias al intercambio de mensajes de vídeo entre el personal sanitario y los padres, se proporcionan actualizaciones frecuentes sobre el estado de los recién nacidos ingresados en la Unidad de Cuidados Neonatales, lo que facilita el vínculo cuando no es posible la presencia de los padres.	*Transmisión de información, demostración del comportamiento, ^**^modificación del entorno [físico], regulación de las emociones y ***regulación de las emociones
Sari y Altay, 2020^(32)^	Transición a la parentalidad (períodos prenatal y posnatal) - fase prenatal: mujeres embarazadas de entre 32 y 37 semanas de gestación; fase pos natal: hasta los tres meses	El *sitio web* se presenta en la fase prenatal en el contexto de la atención primaria de salud, y se utiliza durante el embarazo y en el periodo posnatal en el domicilio	*Sitio web*	A través de *la página web,* que se divide en cuatro secciones, de acuerdo con el Modelo de Promoción de la Salud de Pender - "La *alimentación de mi* bebé", *"El baño y el cuidado de mi* bebé", *"El sueño y* la seguridad *de mi* bebé" y *"La comunicación con mi* bebé"-, se ha facilitado información sobre estos temas. Además de lo mencionado anteriormente, a través de este *sitio web,* los padres tienen acceso a vídeos con información y demostraciones sobre los distintos temas, mensajes motivadores y contacto con profesionales de la salud.	Transmisión de información, regulación de las emociones y demostración del comportamiento
Strand *et a/.,* 20221^(33)^	Parentalidad en el contexto de la hospitalización neonatal: padres de bebés prematuros (con una edad gestacional de entre 24 y 34 semanas), en transición hacia el alta hospitalaria	La aplicación de la intervención digital en desarrollo se llevó a cabo tanto en el contexto hospitalario como en el doméstico	Aplicación móvil digital de *eHealth* adaptada a *tabletas Andró id*	A través de la aplicación móvil digital, el personal de enfermería de la Unidad de Cuidados Neonatales puede comunicarse con los padres mediante mensajes de texto, fotografías y videollamadas. Además, a través de la aplicación móvil digital, es posible registrar y monitorizar los datos clínicos del bebé (peso, niveles de oxigenación, ingesta alimentaria), lo que permite a los enfermeros realizar un seguimiento a distancia. Al utilizar esta aplicación, los profesionales sanitarios también pueden observar y evaluar al bebé mediante videollamadas.	Transmisión de información y regulación de las emociones
Di Mauro *et al.,* 2021^(34)^	Parentalidad en el contexto de la hospitalización neonatal: padres de recién nacidos prematuros de hasta 2 años de edad	Difusión en consultas de atención primaria a los 2, 4, 6 y 12 meses de vida y uso en el domicilio	Página en la red social *"FacebooU"*	A través de la página de Facebook*, los padres pueden acceder a contenidos claros y sencillos sobre los riesgos y beneficios de las vacunas, las enfermedades que se previenen con la vacunación y las edades recomendadas para las vacunas según el programa de vacunación italiano.	Difusión de la información
Militello *et al.,* 2021^(33)^	Transición a la parentalidad (periodos prenatal y posnatal): mujeres y familias en la fase perinatal entre las semanas 1 7 y 36 de gestación	Entorno doméstico	Aplicación móvil digital interactiva con voz llamada *"Self-Management Intervention-Life Essentials* (SMILE)"	A través de la aplicación móvil digital, se ofrece información mediante podcasts secuenciales de entre tres y ocho minutos de duración sobre las necesidades y los cuidados del bebé, los cuidados posparto y la depresión posparto, la alimentación del bebé/lactancia materna y estrategias para gestionar la relación de pareja, centrándose en la educación perinatal. Al final de cada minipodcast, los padres transmitían *sus comentarios* sobre cada uno de ellos a través de grabaciones de audio. Además, se proporcionaron enlaces a documentos fiables y una lista de contactos útiles para acceder a recursos profesionales.	Transmisión de información
Chua *et al.,* 2024 ;i!	Transición a la Parentalidad (periodos prenatal y posnatal): padres en el periodo perinatal y posnatal (desde las 24 semanas de gestación hasta un mes después del parto)	Intervención presentada en el ámbito hospitalario con aplicación en el domicilio	Aplicación móvil digital con un *chatbot* denominada "Parentbot": un asistente digital de salud (PDA)	A través de la aplicación móvil digital, se proporcionó a las madres apoyo informativo, psicológico y social desde el embarazo (a partir de las 24 semanas de gestación) hasta el primer mes después del parto. Dicho apoyo se ofrece mediante: Materiales educativos; Un foro de debate y apoyo; Actividades para la regulación emocional; *Un chatbot* integrado.	Transmisión de información, regulación de las emociones y modificación del entorno [social]
Myneni *et al.,* 2024^(37)^	Etapa perinatal en mujeres con embarazos de alto riesgo (diabetes gestacional, hipertensión y depresión peri parto)	Utilizada durante el embarazo en el contexto ambulatorio	Dos aplicaciones digitales: *"MyPregnancyChart" y ^**^"MomMind"	A través de la aplicación *MyPregnancyChart,* las mujeres embarazadas pueden abordar diversos temas en un foro de debate *en línea* y registrar datos clínicos, lo que permite que el sistema avise cuando sea aconsejable una evaluación médica, atendiendo así a la vigilancia, el mantenimiento y/o el cambio de comportamiento. A través de la aplicación *MomMind,* las madres tienen acceso a un repositorio categorizado por temas que ayuda a identificar los síntomas de la depresión, enseña estrategias para lidiar con el estrés, la tristeza o la ansiedad, y reconoce y normaliza las dificultades emocionales que surgen en el periodo perinatal.	*Modificación del entorno [social], ^**^transmisión de información y ^**^regulación de las emociones

Con el fin de profundizar en la claridad analítica de esta revisión de alcance, las intervenciones educativas que utilizan tecnologías digitales se sistematizaron a través de cuatro técnicas de cambio conductual —transmisión de información, demostración del comportamiento, modificación del entorno y regulación de las emociones—, lo que permitió poner de manifiesto cómo estos elementos contribuyen al *empowerment* parental en situaciones de vulnerabilidad, tal y como se muestra en la Figura 2.

**Figura 2:** Técnicas de cambio conductual utilizadas en las intervenciones educativas digitales y su contribución al*empowerment* parental en situaciones de vulnerabilidad – Lisboa, Portugal, 2025.

## DISCUSIÓN

### Situaciones de Vulnerabilidad en la Parentalidad

En el marco de esta revisión de alcance, la vulnerabilidad en la parentalidad se entiende como una condición dinámica y multidimensional, resultado de la interacción entre factores individuales, clínicos, psicosociales y contextuales, que pueden comprometer la capacidad de los padres para ejercer su parentalidad de manera informada, segura y con confianza. Estas situaciones incluyen a padres y familias que, en determinados momentos del ciclo vital o en contextos específicos de atención de la salud, enfrentan exigencias adicionales, incertidumbre, estrés emocional o limitaciones en el acceso a recursos y apoyo profesional.

Los estudios incluidos evidencian que la vulnerabilidad en la parentalidad surge, sobre todo, en períodos de transición y adaptación, así como en contextos clínicos complejos, en los que los padres se enfrentan a nuevas responsabilidades, decisiones críticas y necesidades de aprendizaje intensivo.

Las situaciones de vulnerabilidad descritas en los estudios incluidos se centran en cuatro ejes: (1) transición a la parentalidad (período prenatal y posnatal); (2) posparto y adaptación a la parentalidad; (3) embarazo de alto riesgo y vulnerabilidad clínica materna; y (4) parentalidad en contexto de hospitalización neonatal. En general, las poblaciones abarcan el período gestacional, el posparto y el período neonatal, extendiéndose hasta la primera infancia, hasta los 36 meses, etapa reconocida como crítica para el desarrollo infantil y para la consolidación de las competencias parentales.

En lo que respecta a la transición a la parentalidad, con enfoque en los períodos prenatal y posnatal, cuatro estudios se centraron en el período gestacional: mujeres entre las semanas 17 y 36^(35)^; padres a partir de las 24 semanas^(36)^; y mujeres embarazadas entre las semanas 32 y 37^(32,37)^. En estos contextos, la vulnerabilidad puede asociarse a la anticipación del ejercicio de la parentalidad, a la necesidad de adquirir conocimientos y competencias, y a la gestión de las expectativas y emociones ante la inminente parentalidad.

En cuanto al posparto y la adaptación a la parentalidad, cinco estudios incluyeron el período posnatal^(28,29,30,31,32,36)^, con seguimiento hasta un mes^(36)^, seis meses^(30)^ y un año^(28)^. Dos estudios se centraron específicamente en madres hasta tres meses^(29)^ y siete meses^(32)^. En estos casos, la vulnerabilidad puede surgir de la sobrecarga física y emocional, de las dificultades para adaptarse al nuevo rol y de la necesidad de apoyo continuo en el manejo de los cuidados del recién nacido.

Solo un estudio se centró en el embarazo de alto riesgo, dirigido específicamente a mujeres con hipertensión/diabetes gestacional y depresión periparto^(37)^.

En relación con la parentalidad en el contexto de la hospitalización neonatal, se identificaron tres estudios, que se desarrollaron con: padres de recién nacidos ingresados en la UCIN^(31)^; padres de bebés prematuros (24–34 semanas) en transición hacia el alta^(33)^; y padres de recién nacidos pretérmino hasta los 2 años^(34)^. En conjunto, estos estudios parecen evidenciar una vulnerabilidad prolongada asociada a la prematuridad, la hospitalización y la continuidad de la atención tras el alta, caracterizada por la ansiedad, la necesidad de información especializada y el apoyo en la adquisición de competencias parentales en contextos altamente tecnificados.

### Contexto de Aplicación de la Intervención Educativa con uso de Tecnologías Digitales

En consonancia con el objetivo de esta revisión de alcance de mapear los contextos de aplicación de las intervenciones educativas digitales que promueven el *empowerment* parental, los resultados revelan que estas intervenciones se desarrollan en diferentes contextos, lo que refleja la diversidad de necesidades de las familias y la flexibilidad de estas herramientas para adaptarse a diferentes entornos.

En los estudios incluidos, se describen intervenciones que utilizan tecnologías digitales presentadas en el contexto de la atención de la salud y utilizadas por la población objetivo en el contexto hospitalario y/o domiciliario. Por ejemplo, tres estudios describen intervenciones que utilizan tecnologías digitales que, aunque se inician en el ámbito hospitalario, también están diseñadas para su uso en el hogar, promoviendo la continuidad de la atención más allá del alta^(30,31,33,36)^ *.* Uno de estos estudios describe no solo la posibilidad del uso domiciliario de la aplicación IFDC, sino también soluciones exclusivamente hospitalarias, como audífonos con cancelación de ruido y acceso biométrico a la UCN^(31)^.

Otros estudios describen intervenciones educativas diseñadas para uso en el hogar, aunque presentadas en un contexto clínico. Entre los ejemplos se incluyen intervenciones introducidas por equipos hospitalarios^(30,36)^ en la atención primaria^(32,34)^ o en consultas ambulatorias^(37)^ para su posterior uso por parte de los padres en el hogar. Estos resultados ponen de manifiesto el potencial de las tecnologías digitales como posibles mediadores de la transición entre contextos de atención. En particular, en uno de ellos, los autores describieron la tecnología utilizada durante las consultas realizadas a los dos, cuatro, seis y 12 meses de vida del niño^(34)^.

### Intervenciones Educativas con Recurso a Tecnologías Digitales

Los estudios incluidos abordaron diversas tecnologías digitales como recurso para promover el *empowerment* parental en contextos de vulnerabilidad. Las aplicaciones se destacaron como el recurso más frecuente, presentes en cinco artículos^(29,30,31,33,35,36,37)^, con funcionalidades que variaron entre *eHealth* ^(33)^, asistente de voz^(35)^, *chatbot* de apoyo parental^(36)^, apoyo a familias en la UCN^(31)^ y aplicaciones para información y apoyo emocional a las madres^(37)^.

La segunda tecnología digital más utilizada en los estudios incluidos fueron las plataformas digitales y los *sitios web* ^(28,29,32,34)^. Destacan una página en la red social Facebook^®(34)^ y *sitios web* educativos de navegación intuitiva^(28,29,32)^. Esta diversidad de recursos tecnológicos pone de manifiesto diferentes estrategias de acceso a la información y de apoyo parental, en función del contexto y de las características de la población objetivo.

Algunos estudios recurrieron además a otras tecnologías digitales, como el envío de mensajes de texto, llamadas telefónicas^(30)^ o el uso de un sistema de videomensajes para facilitar la comunicación entre los padres y el equipo de salud^(31)^. Dos tecnologías digitales complementarias identificadas durante este proceso fueron el acceso libre a la UCN por parte de los padres mediante huella digital y el uso de *headphones* con cancelación de ruido^(31)^. A pesar de la diversidad de recursos tecnológicos, los estudios coinciden en valorar la accesibilidad, la interactividad y la personalización como elementos clave para el éxito de las intervenciones digitales de apoyo a la parentalidad en contextos de vulnerabilidad.

### Intervenciones Educativas con uso de Tecnologías Digitales para Promover el Empowerment en Materia de Parentalidad en Situaciones de Vulnerabilidad

Las intervenciones educativas que utilizan tecnologías digitales para promover el *empowerment* parental en situaciones de vulnerabilidad identificadas en esta revisión se clasificaron en cuatro categorías, correspondientes a las técnicas de cambio conductual aplicadas: (i) transmisión de información; (ii) demostración del comportamiento; (iii) modificación del entorno; y (iv) regulación de las emociones^(9)^.

La mayoría de las intervenciones se agrupan en la transmisión de información, es decir, se centran en promover el conocimiento de los padres sobre conceptos y temas esenciales que deben desarrollarse y adquirirse en la parentalidad^(28,29,30,31,32,33,34,35,36,37)^. De estas intervenciones, solo dos presentan la transmisión de información como la única y exclusiva forma de promover el *empowerment* parental^(34,35)^.

Cuatro de los estudios identificados combinan la transmisión de información con la regulación de las emociones, recurriendo a estrategias diseñadas para reducir la ansiedad/miedo y fortalecer la autoeficacia^(30,32,36,37)^. En otro estudio, una intervención predominantemente informativa pareció contribuir, de manera indirecta y consecuente, a la regulación emocional de los padres, quienes reportaron mayor autoeficacia, seguridad y confianza en su desempeño parental^(33)^. Estos hallazgos evidencian la interrelación entre los componentes informativos y emocionales en el proceso de *empowerment* parental.

Un estudio describe una intervención digital que promueve el *empowerment* parental exclusivamente a través de la regulación de las emociones, utilizando una plataforma en línea para el envío de mensajes informativos y de refuerzo positivo, además de contenido multimedia sobre el estado y el desarrollo del recién nacido, lo que permite el seguimiento de los hitos significativos a distancia^(31)^.

La demostración del comportamiento aparece en dos intervenciones – un sitio web^(32)^ y la aplicación “*Integrated Family Delivered Care*”^(31)^ -, que ofrecen videos demostrativos de los cuidados, además de contenidos informativos y componentes de regulación emocional.

Cabe destacar que seis de las 14 intervenciones promueven el *empowerment* parental a través de la modificación del entorno: cuatro a nivel del entorno social^(28,29,36,37)^ – mediante la creación de foros de discusión en línea entre padres – y dos en el entorno físico – mediante el uso de audífonos^(31)^ con cancelación de ruido para permanecer en la UCN y el acceso mediante huella digital^(31)^ a la unidad. Estas estrategias contribuyeron también a la regulación de las emociones (reducción de la ansiedad de separación y de la angustia asociada a las dificultades de acceso).

En resumen, los resultados parecen evidenciar que, mientras que algunas intervenciones operan a través de un único componente conductual, otras combinan múltiples categorías, a menudo interrelacionadas y con efectos consecuentes sobre el *empowerment* parental, respondiendo de manera diferenciada a las situaciones de vulnerabilidad identificadas.

## CONCLUSIÓN

Esta revisión de alcance ha permitido mapear las intervenciones educativas que utilizan tecnologías digitales para promover el *empowerment* parental en situaciones de vulnerabilidad, poniendo de manifiesto la diversidad de contextos, recursos tecnológicos y técnicas de cambio conductual movilizadas a lo largo de la transición hacia la parentalidad y durante su ejercicio.

Los resultados sugieren que las tecnologías digitales constituyen recursos particularmente relevantes en contextos de vulnerabilidad, al permitir responder, de manera flexible, continua y personalizada, a las necesidades informativas y emocionales de los padres, especialmente en los períodos prenatal y posnatal y en contextos de hospitalización neonatal. En este sentido, el *empowerment* parental surge no solo como un resultado esperado, sino como un proceso dinámico, sustentado por la combinación de información basada en la evidencia, apoyo emocional y modificación del entorno de cuidados.

Las intervenciones educativas digitales identificadas fueron en su mayoría mediadas por enfermeros, lo que refuerza la intervención de estos profesionales como agentes centrales en la promoción de la parentalidad, la alfabetización en salud y la autonomía parental, en consonancia con un enfoque centrado en la familia. La integración de estas intervenciones parece poder ampliar el alcance de la intervención educativa más allá del contacto presencial, favoreciendo la continuidad de la atención y el fortalecimiento de la relación y la comunicación entre padres y profesionales de la salud.

La principal contribución de esta revisión reside en la organización de las intervenciones educativas digitales según técnicas de cambio conductual, lo que permite una lectura más explicativa sobre los mecanismos a través de los cuales estas intervenciones pueden promover el *empowerment* parental en situaciones de vulnerabilidad.

A pesar del potencial identificado, persisten lagunas relevantes en la literatura, en particular la escasez de estudios con diseño experimental o cuasi-experimental, la evaluación limitada del impacto a mediano y largo plazo de las intervenciones y la escasa exploración de la perspectiva de los profesionales de la salud involucrados en su implementación. Se sugiere que las investigaciones futuras den prioridad al desarrollo y la evaluación de intervenciones digitales diseñadas conjuntamente con padres y profesionales, integrando múltiples técnicas de cambio conductual y evaluando resultados centrados en el *empowerment* parental y en los procesos de transición hacia y durante la parentalidad.

Esta revisión no incluyó la evaluación de la calidad metodológica de los estudios, de conformidad con la metodología del JBI para revisiones de alcance, lo que limita las inferencias sobre la solidez de los efectos.

Aunque se llevó a cabo una búsqueda exhaustiva en múltiples bases de datos, la ausencia de una búsqueda sistemática en bases de datos internacionales de tesis y disertaciones, y la limitación de la búsqueda en *Google Scholar* a las primeras diez páginas de resultados, pueden haber condicionado la identificación de toda la evidencia potencialmente relevante. Estos aspectos refuerzan la necesidad de estudios primarios metodológicamente rigurosos y de futuras revisiones sistemáticas que profundicen en la evaluación de la efectividad de las intervenciones educativas digitales que promueven el e*mpowerment* parental.

## Data Availability

Todo el conjunto de datos que apoya los resultados de este estudio fue publicado en el propio artículo.
